# Cultured bacteria isolated from primary sclerosing cholangitis patient bile induce inflammation and cell death

**DOI:** 10.1128/msphere.00550-25

**Published:** 2025-10-20

**Authors:** Chelsea E. Powell, Megan D. McCurry, Silvia Fernanda Quevedo, Lindsay Ventura, Kumar Krishnan, Malav Dave, Shaikh Danish Mahmood, Katherine Specht, Raghav Bordia, Molly R. Sargen, Daniel S. Pratt, Joshua R. Korzenik, A. Sloan Devlin

**Affiliations:** 1 Department of Biological Chemistry and Molecular Pharmacology, Harvard Medical School 1811 , Boston, Massachusetts, USA; 2 Division of Gastroenterology, Hepatology and Endoscopy, Brigham & Women’s Hospital, Boston, Massachusetts, USA; 3 Autoimmune and Cholestatic Liver Center, Massachusetts General Hospital 2348, Boston, Massachusetts, USA; 4 Department of Microbiology, Harvard Medical School 1811 , Boston, Massachusetts, USA; University of Michigan Medical School, Ann Arbor, Michigan, USA

**Keywords:** primary sclerosing cholangitis, bacterial isolates, biliary microbiome, cellular assays, cellular phenotype

## Abstract

Primary sclerosing cholangitis (PSC) is a chronic liver disease characterized by inflammation and progressive fibrosis of the biliary tree. PSC pathogenesis remains poorly understood, and there are no effective therapies. Previous studies have observed associations between colonic and biliary microbiome alterations and PSC. We aimed to determine whether bacterial isolates cultured from PSC patient bile induce disease-associated phenotypes in cells, specifically cell death, epithelial permeability, inflammation, and changes in host-protective pathways. Bile was collected from PSC patients by endoscopic retrograde cholangiography and from non-PSC controls undergoing cholecystectomies. Biliary bacteria were cultured anaerobically, and 50 colonies per sample were identified by 16S sequencing. No bacteria were isolated from non-PSC controls, while bacteria were cultured from most PSC patients. The PSC bile microbiomes exhibited reduced diversity compared to the gut or oral cavity, with one or two species predominating. The effects of supernatants from seven PSC-associated bacterial isolates on cellular phenotypes were characterized using human colonic (Caco-2), hepatic (HepG2), and biliary (EGI-1) cells. Overall, PSC-associated bacteria produced factors cytotoxic to hepatic and biliary cells. An *Enterococcus faecalis* isolate, and to a lesser extent a *Veillonella parvula* isolate, induced epithelial permeability, while *Escherichia coli*, *Fusobacterium necrophorum*, and *Klebsiella pneumoniae* isolates induced inflammatory cytokines in biliary cells. Our data suggest that bacteria cultured from PSC bile induce cellular changes characteristic of PSC pathogenesis, with different isolates inducing distinct cellular responses. Our work provides a starting point for future research into bacterial contributions to PSC with the eventual goal of developing therapies for this disease.

**IMPORTANCE:**

Primary sclerosing cholangitis (PSC) is a chronic liver disease in which inflammation and scarring of the bile ducts cause bile to build up in the liver, leading to liver damage and eventually liver failure. The causes of this disease are poorly understood, and the only current treatment is a liver transplant. To develop new treatments, we must first better understand what leads to this disease. We examined whether bacteria isolated from PSC patient bile can cause disease-related responses in human biliary, liver, and intestinal cells. We observed that different PSC-associated bacteria can induce distinct disease-related cellular changes, including inflammation and cell death. These data suggest that the microbial community in PSC patients may indeed be linked to disease development. Our findings provide new starting points for further exploration into the poorly understood origins of PSC.

## INTRODUCTION

Primary sclerosing cholangitis (PSC) is a rare, chronic disorder characterized by inflammation and progressive fibrosis of the biliary ducts, which can lead to the development of multifocal biliary strictures, secondary biliary cirrhosis, and decompensated liver disease (1). PSC is closely linked with inflammatory bowel disease (IBD); approximately 70%–80% of PSC patients have also been diagnosed with IBD (2, 3). PSC is also associated with a higher risk of cancer in the biliary tract and colon (1). Currently, there are no effective treatments for PSC other than liver transplant, and PSC can still recur after transplant (4, 5). The pathophysiology of PSC is poorly understood. Studies into the mechanisms underlying PSC disease phenotypes are essential for the development of therapies beyond liver transplantation.

While the origins of PSC are unknown, it is currently thought that a combination of environmental and genetic factors contributes to PSC pathogenesis (2, 6). Among environmental triggers, the gut microbiota is hypothesized to play a key role in PSC development, a theory supported by alterations in microbial community composition detailed in both PSC and IBD (2). Previous studies have profiled the gut (7–11) and biliary (12–14) microbiome of PSC patients compared to a variety of non-PSC controls. Studies of the gut microbiome in PSC have overall identified lower microbial diversity in PSC patients compared to non-PSC controls (8–11). Two previous studies of the biliary microbiome identified lower microbial diversity in PSC patients compared to non-PSC controls undergoing endoscopic retrograde cholangiography (ERCP) (12, 13). However, the biliary microbiome is currently understudied, and whether or not bile is sterile in healthy livers remains a topic of debate (15, 16). The gut and biliary microbiomes of PSC patients have been shown to contain an overrepresentation of certain bacterial taxa compared to controls. While the methodologies of these studies were diverse, *Veillonella*, *Streptococcus*, *Enterococcus*, and *Fusobacterium* were more abundant in PSC patients consistently across studies (2, 7). Previous studies have also included fecal (10) or plasma (9) metabolomics and observed changes in the abundance of microbiota-derived metabolites in PSC patients compared to controls. Taken together, these alterations in microbial populations and metabolite production suggest that the microbiome may be playing a functional role in the development of PSC. While some studies have used mouse models to explore the therapeutic potential of targeting PSC-derived *Klebsiella pneumoniae* (17, 18), the direct relationship between specific PSC-associated bacteria and cellular phenotypes remains underexplored.

Given how little is known about the causal roles that bacteria may play in PSC pathogenesis, we decided to investigate whether isolating bacteria from PSC patients could provide a starting point for researchers to investigate the underlying causes of this disease. As a first step down this path, in this work, we isolated bacteria from PSC patient bile to characterize the ability of individual isolates to induce disease-associated cellular responses in human colonic, hepatic, and biliary cells, including cell death, epithelial barrier damage, and inflammation. Both our microbial culturing and cellular assays were performed anaerobically to mimic the oxygen-limited environment of the biliary tree in PSC. From the seven PSC-derived isolates profiled, we determined that while PSC-associated bacteria in general appeared to produce factors that are cytotoxic to hepatic and biliary cells, other cellular phenotypes were more specific to certain isolates. Only an *Enterococcus faecalis* isolate, and to a lesser extent a *Veillonella parvula* isolate, could induce epithelial permeability, while *Escherichia coli*, *Fusobacterium necrophorum*, and *Klebsiella pneumoniae* isolates induced the expression of genes encoding inflammatory cytokines in biliary cells. These results show that bacteria in PSC patient bile can cause inflammation and cellular damage and suggest that different bacterial species may contribute to distinct aspects of disease pathogenesis. Our findings provide proof of concept that further mechanistic studies of PSC-associated bacteria are a viable avenue for better understanding the causes of this disease.

## RESULTS

### PSC patient bile contains anaerobically culturable bacteria

**Fig 1:**
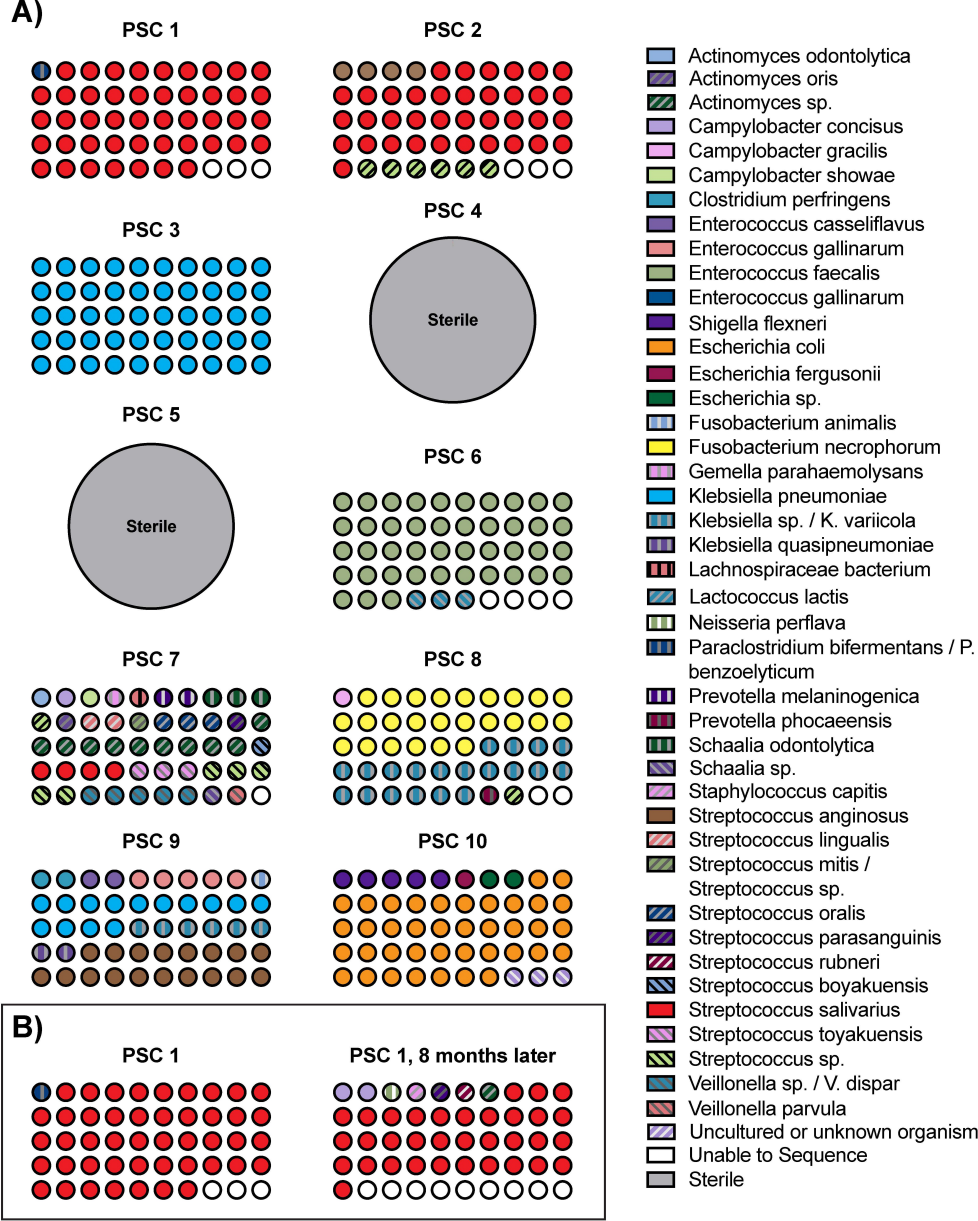
PSC patient bile contains culturable bacterial isolates. (**A**) Fifty bacterial colonies were isolated from each distinct patient’s bile sample under anaerobic conditions. Bacteria were identified by 16S rRNA gene PCR and Sanger sequencing. 〇 = 1 colony out of 50. (**B**) Anaerobically culturable bacteria were isolated from bile samples from the same patient, 8 months apart. Note that the figure is meant to depict an overall visual representation of the diversity of bacterial species within each sample. For complete lists of cultured bacterial species and clonality groups (where appropriate) in each sample, see Tables S2 to S10.

We collected bile by ERCP from 10 individuals with PSC as well as from three individuals undergoing cholecystectomies without PSC who served as control subjects (Table 1; Table S1). We cultured bile under anaerobic conditions because fibrotic liver diseases are associated with chronic hypoxia that may select for anaerobic bacteria (19, 20). We observed that the majority of the PSC patient bile samples contained culturable bacteria. By contrast, none of the control samples contained culturable microbes (Table 1). Colony picking followed by 16S rRNA sequencing of restreaked isolates further revealed that most of the bile samples of PSC patients were dominated by 1–2 species of bacteria (Fig. 1). Two bile samples taken from the same patient 8 months apart demonstrated that it is possible for the same 1–2 species to dominate the biliary microbiome in the same individual over time (Fig. 1B). These isolates were also examined for their ability to produce H_2_S, which has been linked to IBD, particularly ulcerative colitis (UC) (21–25). Of the PSC samples with culturable bacteria, half (4 of 8) contained H_2_S-producing isolates (Tables S2 to S10). Metagenomic analysis, while not possible for all samples due to the difficulty of gDNA extraction from bile (see Materials and Methods), generally validated our PSC bile culturing results. Most samples exhibited a similar overrepresentation of the same 1–2 species of bacteria that we observed in our bacterial culturing (Fig. S1). However, metagenomic analysis of bile samples from PSC 4 and PSC 9 did reveal the presence of several uncultured species. Overall, these results show that there were live bacteria in the bile from the majority of PSC patients in this cohort. While most samples contained one or two dominant species, the identity of these species differed from patient to patient, highlighting the heterogeneity of individual PSC biliary microbiomes.

**TABLE 1:** Patient characteristics at time of bile collection^*a*^

Characteristic	PSC	Controls
Patients, *n*	10	3
Age (years), median (range)	39.5 (28–73)	41 (35–59)
Female, *n* (%)	2 (20)	2 (66.7)
Age at diagnosis (years), median (range)	34.5 (23–70)	NA
Disease duration (years), median (range)	8.5 (0–27)	NA
IBD, *n* (%)	8 (80)	0
Age at IBD diagnosis (years), median (range)	26 (16–48)	NA
IBD duration (years), median (range)	14 (3–55)	NA
Other inflammatory conditions, *n* (%)	1 (10)	1 (33.3)
ERCP (#), median (range)	4.5 (1–17)	0
ALP (U/L), median (range)	380.5 (58–629)	86 (41–106)
Antibiotic use 3 months prior to collection, *n* (%)	8 (80)	1 (33.3)
Antibiotics on day of collection, *n* (%)	10 (100)	3 (100)
Corticosteroids, *n* (%)	7 (70)	0
5-ASA, *n* (%)	5 (50)	0
Biologics, *n* (%)	2 (20)	0
UCDA, *n* (%)	6 (60)	0
Proton pump inhibitors, *n* (%)	2 (20)	0
Cultured bacterial species, median (range)	2 (0–23)	0 (0–0)

^*a*^ Acronyms: 5-ASA, 5-aminosalicylic acid; ALP, alkaline phosphatase; ERCP, endoscopic retrograde cholangiography; IBD, inflammatory bowel disease; UCDA, ursodeoxycholic acid; NA, not applicable.

Next, we performed clonality analysis on species within a patient sample for which there were 10 or more isolates of that species. We observed that in most cases, the isolates within a species belonged to a single clone, although there were selected cases where two clones of a species were identified within a patient (i.e., PSC 1–8 months, *Streptococcus salivarius*; PSC 3, *Klebsiella pneumoniae*; PSC 9, *Klebsiella pneumoniae*) (Tables S2 to S10). An important caveat to this analysis is that we trimmed the 16S sequences prior to alignment for clonality. Thus, some base pair differences may have been lost, and we cannot definitively state that there are no differences between the clonal groups identified here. Nonetheless, our results suggest that overall, there appears to be a reasonably high degree of within-species homogeneity in a given patient.

### PSC bacterial isolates produce factors that affect human cell viability

Seven bacterial isolates generated from PSC bile culturing were selected for further characterization using human cellular assays (Table S11). These representative isolates were selected due to the overrepresentation of the species to which they belonged in PSC patient bile in our study (*Enterococcus faecalis*, *Escherichia coli*, *Fusobacterium necrophorum*, *Klebsiella pneumoniae*, and *Streptococcus salivarius*) or because the species were identified as abundant in PSC patient microbiomes in previous studies (*Veillonella dispar* and *Veillonella parvula*) (8, 10, 11, 26, 27). *Bacteroides fragilis* ATCC 25285 (hereafter called *B. fragilis*) (28), a human gut commensal strain that has been shown to promote epithelial integrity, and *Enterococcus gallinarum* (isolated by the Kriegel lab at Yale School of Medicine, hereafter called *E. gallinarum*), a pathogenic strain that has been shown to induce intestinal permeability and translocate from the gut to the liver (29), were selected for comparison to the PSC-derived isolates. Importantly, *B. fragilis* ATCC 25285 is an enterotoxin-free strain that has been characterized as a protective probiotic (28, 30) and is distinct from the subset of enterotoxigenic *B. fragilis* associated with UC (31, 32).

**Fig 2:**
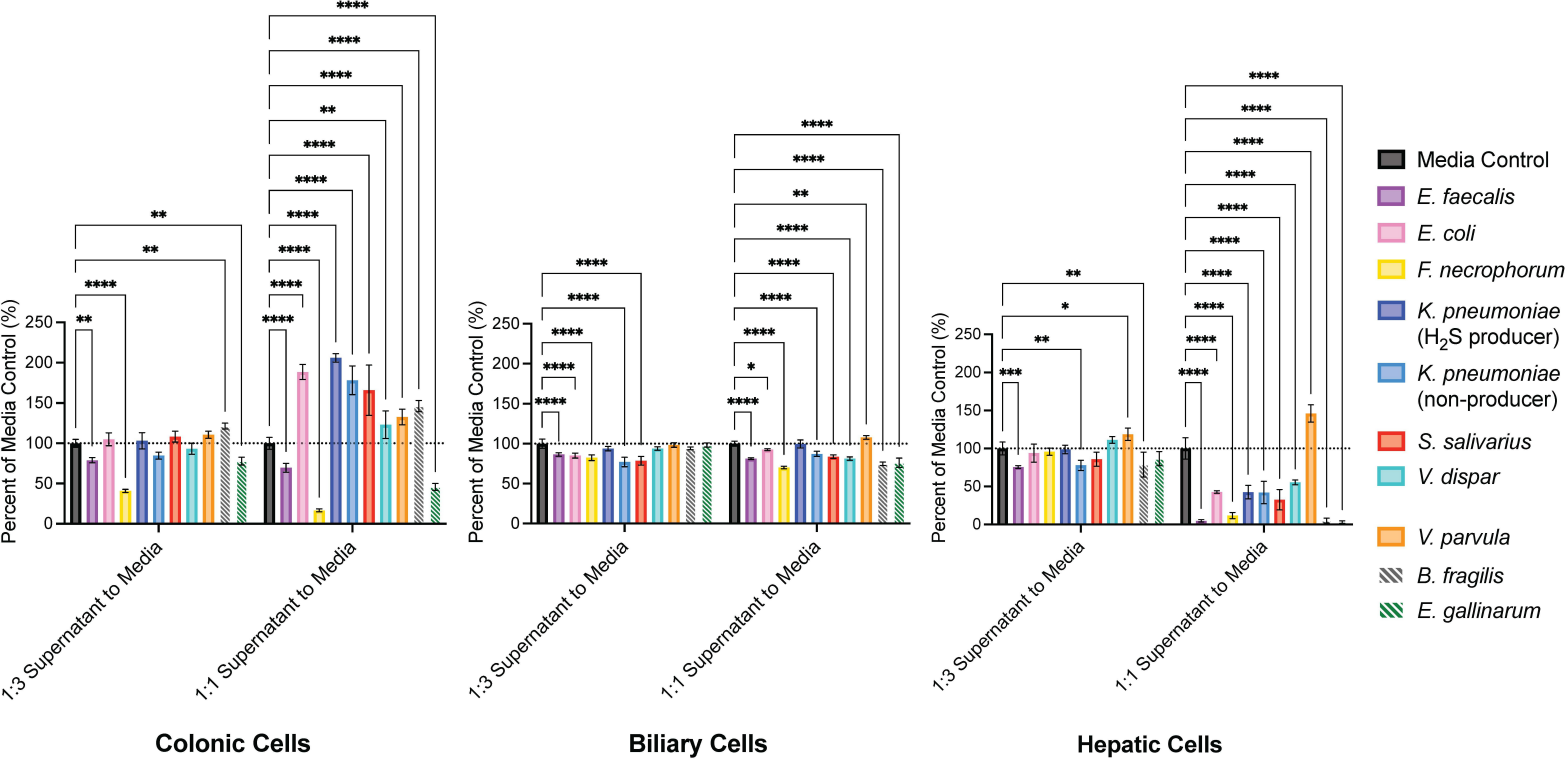
PSC bacterial supernatants affect human cell viability. Colonic (Caco-2), biliary (EGI-1), and hepatic (HepG2) human cancer cells were treated with a 1:3 or 1:1 ratio (vol/vol) of bacterial culture supernatant for 16 h under anaerobic conditions. BHI+ medium was used as a control. *B. fragilis* and *E. gallinarum* were used as reference strains and were not derived from PSC patients, as indicated by striped bars. The following isolates were used in these experiments (Table S11): *E. faecalis*_PSC06_col01, *E. coli*_PSC10_col01, *F. necrophorum*_PSC08_col02, *K. pneumoniae*_PSC09_col49 (H_2_S producer), *K. pneumoniae*_PSC03_col01 (non-producer), *S. salivarius*_PSC01_col01, *V. dispar*_PSC07_col14, and *V. parvula*_PSC07_col05. Two-way ANOVA was performed followed by Dunnett’s multiple comparisons test using Graphpad Prism 10 software (values are shown as mean ± SD; four biological replicates; *P < 0.05, **P < 0.01, ***P < 0.001, ****P < 0.0001).

PSC is characterized by biliary epithelial injury and progressive fibrosis (1). We therefore prioritized assessing cell viability to determine whether PSC-associated bacteria produce factors capable of directly damaging hepatic and biliary cells, potentially initiating or perpetuating tissue injury. Because the majority of PSC patients also have inflammatory bowel disease (IBD) and bacterial translocation from the gut to the biliary tree has been proposed as a potential contributor to PSC pathogenesis, we also tested whether bacterial supernatants affected colonic cell viability (2, 3). Cell viability assays were performed using established colonic (Caco-2), biliary (EGI-1), and hepatic (HepG2) human cancer cell lines under anaerobic conditions. The effects on cell viability of gut commensal reference strain *B. fragilis* and pathogenic reference strain *E. gallinarum* were otherwise previously unknown against these specific human cell lines. The gut commensal control, *B. fragilis*, did not induce cell death in the colonic cell line (Fig. 2). *B. fragilis* instead induced growth in Caco-2 cells, and induction of slight growth has indeed been seen in a previous study using normal colonic epithelial cells (hcoEPIC) (28). *E. gallinarum* induced cell death in all three cell lines, while *B. fragilis* induced cell death in the biliary and hepatic cell lines. The fact that *B. fragilis* is a gut commensal that does not usually interact with biliary and hepatic cells may have contributed to its cytotoxic effects on these cell types. Sterile-filtered bacterial supernatants from all but one PSC-derived bacterial isolate induced cell death in both biliary and hepatic cells, with hepatic cells being affected to a greater extent (Fig. 2). Notably, the PSC bacterial supernatants, with the exception of *E. faecalis*_PSC06_col01 and *F. necrophorum*_PSC08_col02, induced cell growth instead of cell death in colonic cells. *V. parvula*_PSC07_col05 also induced growth in hepatic cells and, to a lesser extent, in biliary cells. These results indicate that some biliary bacteria are producing toxic factors that negatively impact viability in biliary and hepatic cells, but not colonic cells, suggesting that these bacterial factors exert cell-specific effects.

### PSC patient-derived *E. faecalis* and *V. parvula* induce intestinal permeability

**Fig 3:**
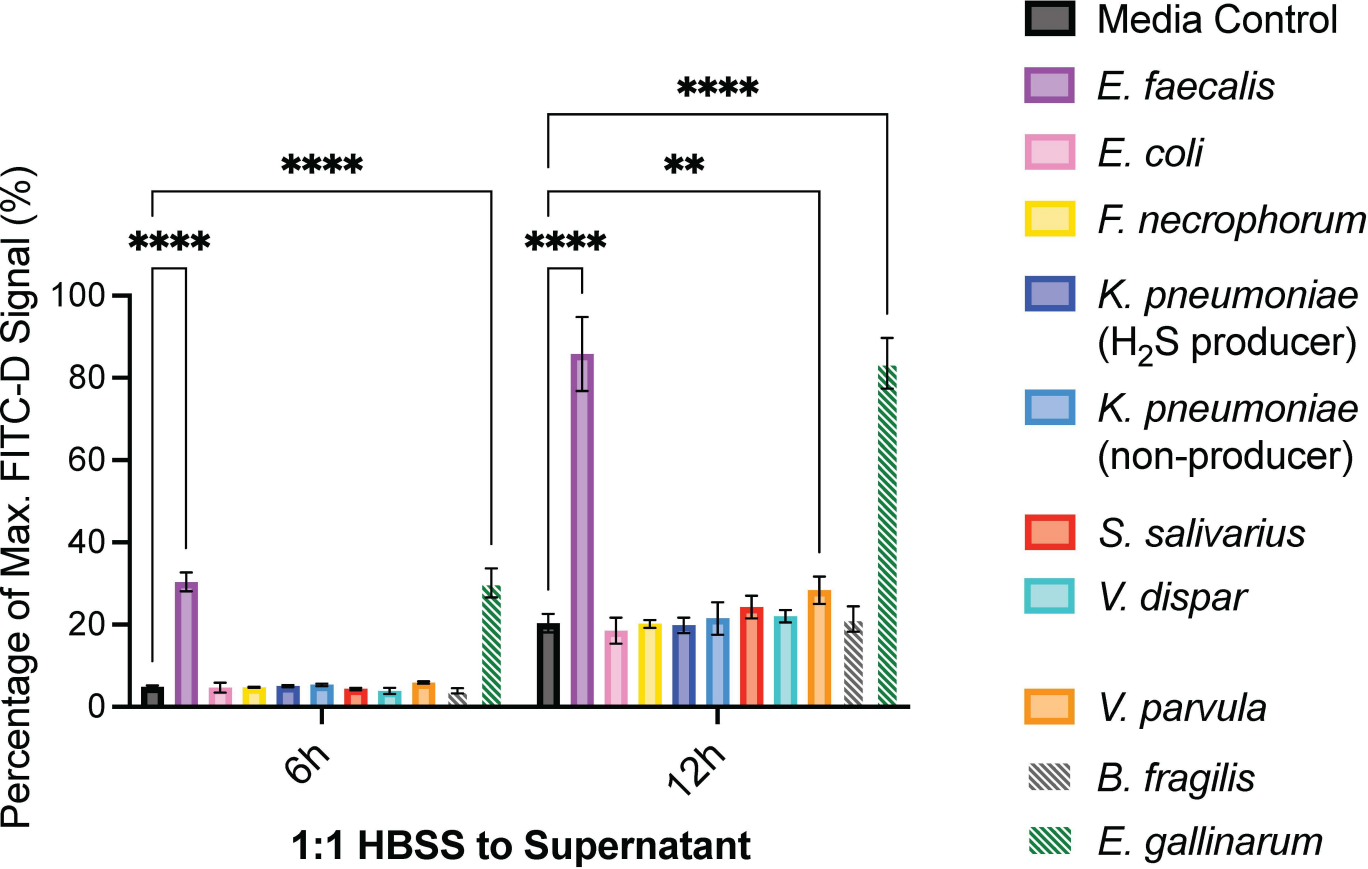
Bacterial supernatants from the PSC patient isolates *E. faecalis* and *V. parvula* increased epithelial permeability. A differentiated monolayer of Caco-2 cells was treated with a 1:1 ratio (vol/vol) of HBSS buffer to bacterial culture supernatant for 6 or 12 h under anaerobic conditions. BHI+ medium was used as a control. *B. fragilis* and *E. gallinarum* were used as reference strains and were not derived from PSC patients, as indicated by striped bars. The following isolates were used in these experiments (Table S11): *E. faecalis*_PSC06_col01, *E. coli*_PSC10_col01, *F. necrophorum*_PSC08_col02, *K. pneumoniae*_PSC09_col49 (H_2_S producer), *K. pneumoniae*_PSC03_col01 (non-producer), *S. salivarius*_PSC01_col01, *V. dispar*_PSC07_col14, and *V. parvula*_PSC07_col05. Two-way ANOVA was performed followed by Dunnett’s multiple comparisons test using Graphpad Prism 10 software (values are shown as mean ± SD; four biological replicates; *P < 0.05, **P < 0.01, ***P < 0.001, ****P < 0.0001).

Increased gut permeability is a hallmark of PSC-IBD and may facilitate translocation of bacteria to the biliary tree, contributing to disease initiation or progression (2, 17). We used a Caco-2 transwell assay to model induction of pathogenic intestinal permeability (33, 34). Reference strain *B. fragilis* did not induce permeability, while reference strain *E. gallinarum* caused barrier damage, as expected from previous studies (28, 29, 35). Of all the PSC-associated bacteria examined, only supernatant derived from *E. faecalis*_PSC06_col01 cultures induced epithelial permeability after both 6 and 12 h of treatment. *V. parvula*_PSC07_col05 supernatant induced permeability at 12 h alone (Fig. 3). Taken together with the colonic cell viability data, these results suggest that *E. faecalis*_PSC06_col01 can contribute to both epithelial cell death and barrier damage, while an array of PSC-associated bacteria may be causing cell damage in the biliary tree and liver.

### PSC bacterial isolates induce inflammatory cytokines in biliary cells

**Fig 4:**
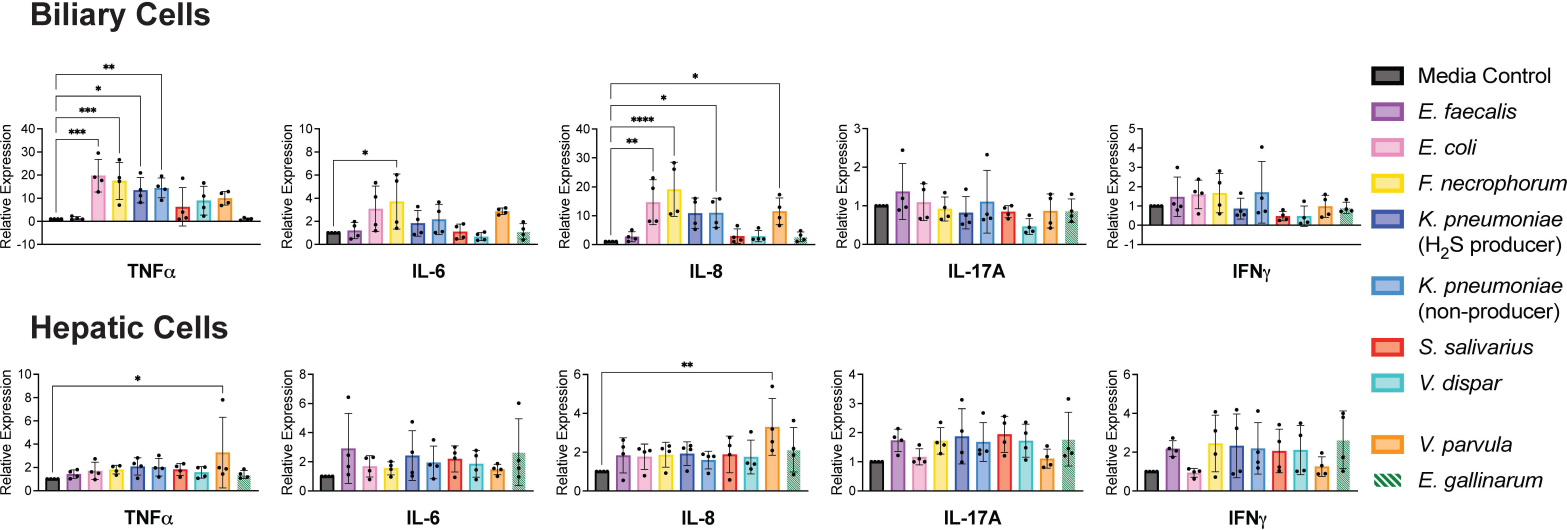
PSC bacterial supernatants induced transcription of inflammatory cytokines in biliary and hepatic cells. Biliary (EGI-1) and hepatic (HepG2) cells were treated with a 1:1 ratio (vol/vol) of bacterial culture supernatant to cell culture medium for 6 h under anaerobic conditions, and the effect on transcription of target genes was assessed by qPCR. BHI+ medium was used as a control. *E. gallinarum* was used as a reference strain and was not derived from PSC patients, as indicated by striped bars. Results were normalized to UBC mRNA expression. The following isolates were used in these experiments (Table S11): *E. faecalis*_PSC06_col01, *E. coli*_PSC10_col01, *F. necrophorum*_PSC08_col02, *K. pneumoniae*_PSC09_col49 (H_2_S producer), *K. pneumoniae*_PSC03_col01 (non-producer), *S. salivarius*_PSC01_col01, *V. dispar*_PSC07_col14, and *V. parvula*_PSC07_col05. One-way ANOVA was performed followed by Dunnett’s multiple comparisons test using Graphpad Prism 10 software (values are shown as mean ± SD; four biological replicates from independent experiments with three technical replicates each; *P < 0.05, **P < 0.01, ***P < 0.001, ****P < 0.0001).

Biliary inflammation is a central feature of PSC (1). To assess whether PSC-associated bacteria could directly elicit inflammatory signaling in biliary epithelial cells, we measured cytokine induction. qPCR after 6 h treatment with PSC-derived bacterial supernatant indicated that *E. coli*_PSC10_col01, *F. necrophorum*_PSC08_col02, and *K. pneumoniae*_PSC03_col01 induced transcription of the pro-inflammatory cytokines TNFα and IL-8 in biliary cells but did not exert the same effect in hepatic cells. *V. parvula*_PSC07_col05 induced inflammatory responses in both cell types (Fig. 4). Our results show that a subset of PSC-associated bacteria induce inflammatory responses in biliary cells and identify TNF⍺ and IL-8 as the primary cytokines induced by these isolates.

### PSC-derived bacteria can affect protective pathways in human biliary and hepatic cells

**Fig 5:**
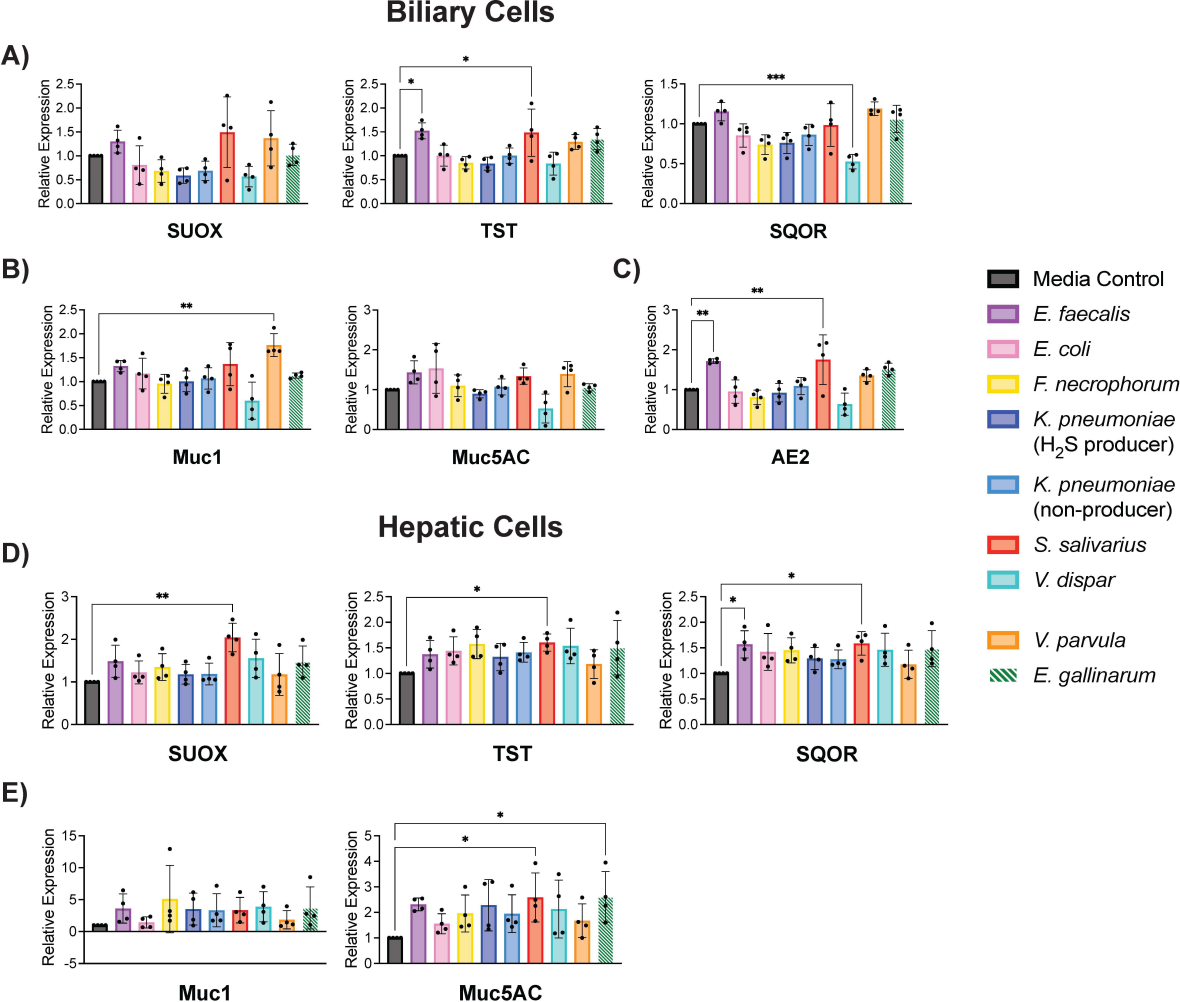
PSC bacterial supernatants affected transcription of genes related to sulfur metabolism, mucin expression, and the bicarbonate umbrella in biliary and hepatic cells. Biliary (EGI-1) and hepatic (HepG2) cells were treated with a 1:1 ratio (vol/vol) of bacterial culture supernatant to cell culture medium for 6 h under anaerobic conditions, and the effect on transcription of target genes was assessed by qPCR. BHI+ medium was used as a control. *E. gallinarum* was used as a reference strain and was not derived from PSC patients, as indicated by striped bars. Results were normalized to UBC mRNA expression. The following isolates were used in these experiments (Table S11): *E. faecalis*_PSC06_col01, *E. coli*_PSC10_col01, *F. necrophorum*_PSC08_col02, *K. pneumoniae*_PSC09_col49 (H_2_S producer), *K. pneumoniae*_PSC03_col01 (non-producer), *S. salivarius*_PSC01_col01, *V. dispar*_PSC07_col14, and *V. parvula*_PSC07_col05. One-way ANOVA was performed followed by Dunnett’s multiple comparisons test using Graphpad Prism 10 software (values are shown as mean ± SD; four biological replicates from independent experiments with three technical replicates each; *P < 0.05, **P < 0.01, ***P < 0.001, ****P < 0.0001).

Because disruption of host protective mechanisms such as mucin production (36–38) and bicarbonate secretion (1, 39) is implicated in PSC pathogenesis, we evaluated whether PSC-derived bacteria could alter these pathways in biliary and hepatic cells. We also examined effects on sulfur detoxification as hydrogen sulfide damage is associated with IBD (21, 22). Supernatants from *V. dispar*_PSC07_col14 cultures caused a decrease in mRNA expression of sulfide quinone oxidoreductase (SQOR) in biliary cells, which indicates damage to a pathway that aids in the clearance of toxic sulfides (Fig. 5). *S. salivarius*_PSC01_col01 and *E. faecalis*_PSC06_col01 supernatants upregulated the expression of sulfur metabolism targets in both hepatic and biliary cells, as well as expression of AE2, a primary component of the protective bicarbonate umbrella in biliary cells. *S. salivarius*_PSC01_col01 also upregulated mucin expression in hepatic cells, while *V. parvula*_PSC07_col05 upregulated mucin expression in biliary cells. Increased expression of genes involved in sulfur metabolism, mucin expression, and the bicarbonate umbrella may indicate that hepatic and biliary cells upregulate protective pathways in response to 6 hours of exposure to toxic factors produced by these bacteria.

## DISCUSSION

The pathogenesis of PSC remains poorly understood, and examining potential mechanistic drivers of the disease is essential for developing new treatments. Previous studies have established that PSC patients have altered gut and biliary microbiomes compared to non-PSC control subjects (7–14). Here, as a first step toward understanding the bacterial contributions to PSC pathogenesis, we cultured bacterial isolates from PSC patient bile and subjected human biliary, hepatic, and colonic cells to factors produced by these bacteria. Through these assays, we identified bacterial isolates that induce PSC-associated cellular phenotypes, including epithelial permeability, biliary and hepatic inflammation, and cell death (Table 2). Interestingly, we observed that isolates from different species induce distinct cellular responses. Our results suggest that the overall microbial community in PSC patients may contribute to disease development as opposed to single strains acting as universal drivers of pathogenesis.

**TABLE 2:** Summary of characterization of bacteria isolated from PSC patient bile

Species	Source	Gram +/−	H_2_S producer?	Biliary viability	Hepatic viability	Colonic viability	Intestinal permeability	Biliary inflammation	Hepatic inflammation
*Enterococcus faecalis*	PSC 6, Colony 1	+	No	Killing	Killing	Killing	Induces	No effect	No effect
*Escherichia coli*	PSC 10, Colony 1	–	Yes	Killing	Killing	Induces growth	No effect	Induces	No effect
*Fusobacterium necrophorum*	PSC 8, Colony 2	–	Yes	Killing	Killing	Killing	No effect	Induces	No effect
*Klebsiella pneumoniae*	PSC 9, Colony 49 (H_2_S producer) and PSC 3, Colony 1	–	Mixed	Killing	Killing	Induces growth	No effect	Induces	No effect
*Streptococcus salivarius*	PSC1, Colony 1	–	No	Killing	Killing	Induces growth	No effect	No effect	No effect
*Veillonella dispar*	PSC 7, Colony 14	–	No	Killing	Killing	Induces growth	No effect	No effect	No effect
*Veillonella parvula*	PSC 7, Colony 5	–	No	Induces growth	Induces growth	Induces growth	Induces	Induces	Induces

First, we observed that in most patients from this cohort, one or two bacterial species predominated in individual PSC patient bile (Table 1; Fig. 1). This finding agrees with previous studies that have demonstrated that the PSC gut and biliary microbiomes contain an overabundance of certain species compared to non-PSC controls (2, 12). Culturing of our non-PSC controls requiring cholecystectomies did not yield any bacterial isolates. By contrast, two previous PSC biliary microbiome studies found greater microbial diversity in non-PSC compared to PSC bile (12, 13). However, the non-PSC controls in these previous studies were from patients without PSC who required an ERCP, not cholecystectomy patients. Comparing our results with this previous work highlights the need to further characterize the biliary microbiome in different disease states. Our non-PSC cholecystectomy bile results align more closely with a recent study, which found that bile from gallbladders with no abnormal pathologies, as judged by a pathologist, did not contain culturable microbes (40). By contrast, *Enterococcus faecalis*, *Escherichia coli*, *Fusobacterium necrophorum*, *Klebsiella pneumoniae*, *Streptococcus anginosus*, and *Streptococcus salivarius* were cultured species in PSC bile (Fig. 1). One confounding factor to note is that all of the PSC patients in this study received antibiotics (80% within 3 months of collection and 100% on the day of collection). While clinically necessary and thus unavoidable, antibiotic use may have enriched for certain species. In addition, while BHI+ is a rich medium that has been previously shown to support the growth of a diverse array of anaerobic bacteria (41–43), we only used one type of medium for bacterial isolation. Both factors may have affected our culturing results.

One limitation of our study is the relatively small number of patient samples collected. To put this work in context, this research is not meant to be a comprehensive study of the bacteria present in PSC bile. Rather, our study represents a starting point demonstrating that bacteria cultured from PSC patient bile can cause inflammation and death in biliary and liver cells, as well as epithelial barrier damage. Moreover, despite the limited sample size, our results are consistent with previous PSC microbiome profiling efforts (2, 7), which observed an overabundance of *Enterococcus*, *Fusobacterium*, and *Streptococcus* in PSC patients. Thus, it is likely that our samples are representative of at least a substantial subset of PSC patient microbiomes that have been consistently identified in the literature, with the exception that we did not see an overrepresentation of *Veillonella*, as has been observed in previous studies (8, 10, 11, 26, 27). Importantly, our culturing results demonstrate that live bacteria are present in the bile of PSC patients, a conclusion that is not possible based on sequencing-based analyses alone.

To identify uncultured bacteria in the bile samples, we performed metagenomic sequencing. However, despite multiple attempts using different techniques (see Materials and Methods), gDNA proved difficult to extract from bile, preventing metagenomic analysis of some patient bile samples. Overall, the bacterial identity and diversity of each sample that we observed in our metagenomic analyses were similar to those from our culturing data. These results suggest that the lower representation of *Veillonella* in our isolates compared to previous PSC microbiome studies was not a result of our culturing methods but indeed reflective of our patient population. For two of the sequenced samples (PSC 4 and PSC 9), we identified some bacteria that we were unable to culture, including species from the genera *Sphingomonas*, *Acidovorax*, and *Campylobacter* (Fig. S1). The identification of uncultured bacteria is of particular note for PSC 4, which appeared sterile during our culturing efforts, suggesting that some bacteria were not amenable to our culturing methods. No bacteria were able to be identified by metagenomic analysis in PSC 7 and PSC 1 (8 months later), despite successful culturing in these samples. One possible explanation for the failure of sequencing in these cases is that gDNA extraction resulted in the collection of mainly eukaryotic DNA, and a negligible amount of bacterial DNA was isolated. This was indeed seen with PSC 7 and PSC 1 (8 months later), where there were only 0.4% and 1% bacterial DNA relative abundance, respectively, seen by profiling using Kraken2 (44) followed by KronaTools (45) (Table S12). Our work highlights the difficulty of extracting bacterial DNA from some bile samples and motivates the development of more robust DNA isolation methods, given the heterogeneity of bile sample characteristics, including viscosity and composition.

In cell culture assays, supernatants from bacteria that we had isolated from PSC bile induced cell death in both biliary and hepatic cells, with hepatic cells being affected to a greater extent (Fig. 2). These results may suggest that cells in the biliary tree can tolerate these bacteria, despite the fact that they are producing cytotoxic factors. This resistance to cell death could potentially allow for bacteria to inhabit the biliary tree, leading to a buildup of these cytotoxic factors that can then cause liver damage. Interestingly, the PSC bacterial supernatants, with the exception of *E. faecalis*_PSC06_col01 and *F. necrophorum*_PSC08_col02, induced cell growth in the colonic cancer cell line. These results may indicate that these bacteria produce growth-promoting factors. *V. parvula*_PSC07_col05 was unique among the PSC-derived bacteria in that it did not induce cell death, but instead induced growth in hepatic cells and, to a lesser extent, in biliary cells. Indeed, an increased abundance of *V. parvula* has been correlated with intrahepatic cholangiocarcinoma (46). Future work is needed to identify potential growth-promoting factors produced by PSC-associated bacteria. While human cancer cell lines are established as useful *in vitro* models due to ease of cell culturing, it should be noted that they may be more responsive, particularly to growth-promoting factors, by nature of being cancer-derived and already in a disease state. Therefore, the effects of these bacteria on colonic, hepatic, and biliary cell health in PSC patients may be more subtle. Of note, the reference commensal strain *B. fragilis* (ATCC 25285), which was not derived from PSC patients, induced growth in colonic cells and cell death in hepatic and biliary cells, despite being an enterotoxin-free strain that is generally considered protective and non-pathogenic (Fig. 2) (28, 30, 35, 47, 48). It is possible that these results with *B. fragilis* are reflective of an increased responsiveness in these cancer cell lines.

Our intestinal permeability assay results demonstrate that *E. faecalis*_PSC06_col01 is a strong inducer of epithelial barrier damage, and *V. parvula*_PSC07_col05 is a weaker inducer, while other PSC-derived bacteria did not have this effect (Fig. 3). These data suggest that *Enterococcus* may be a driver of pathogenic intestinal permeability in PSC patients, potentially allowing for gut bacteria to translocate from the gut to the liver via the portal vein and inhabit the biliary tree. These results appear consistent with previous studies that have identified an increase in abundance of *E. faecalis* in PSC ductal bile compared to controls without PSC (12). They are also consistent with prior work showing that *E. faecalis* can increase intestinal permeability through its gelatinase activity (49). While *E. faecalis* is present in human gut microbiomes, including those of PSC patients (27, 50), we do not have paired fecal samples from the PSC patients in this study to confirm whether individuals with *E. faecalis* in their bile also have this microbe in their GI tract. Future studies pairing gut and bile metagenomic sequencing will help reveal connections between bacteria that induce intestinal permeability and live bacteria in the biliary tree. Notably, our results do not reveal whether the bacteria in PSC patient bile are transient or are colonizing the biliary tract long term. Future studies investigating whether PSC-associated bacterial isolates are bile-resistant would help shed light on the potential ability of bacteria to colonize the biliary tract. In addition, future studies may benefit from examining whether *E. faecalis*_PSC06_col01 or other strains of *E. faecalis* induce biliary permeability in mouse models or in *in vitro* models of biliary epithelial permeability.

We identified the PSC-associated bacteria *E. coli*_PSC10_col01, *F. necrophorum*_PSC08_col02, *K. pneumoniae*_PSC09_col49, *K. pneumoniae*_PSC03_col01, and *V. parvula*_PSC07_col05 as inducers of inflammatory cytokine expression in biliary cells (Fig. 4). These bacteria did not have the same effect in hepatic cells, with the exception of *V. parvula*_PSC07_col05. These results are consistent with PSC disease progression, in which inflammation in the biliary tree precedes liver damage (39). Interestingly, *E. faecalis*_PSC06_col01, the primary isolate that induced intestinal permeability, did not induce biliary inflammation according to our transcriptional analyses. These findings suggest that different PSC-associated bacteria may be responsible for unique aspects of cellular responses. In addition, while *E. coli*_PSC10_col01, *F. necrophorum*_PSC08_col02, and *K. pneumoniae*_PSC09_col49 were all H_2_S producers, we did not observe a stronger induction of inflammation by H_2_S-producing *K. pneumoniae*_PSC09_col49 compared to non-producing *K. pneumoniae*_PSC03_col01 (Fig. 4; Table 2). Therefore, although bacterial H_2_S production may be associated with inflammation, as indicated by our results and as suggested by other studies (51), H_2_S production does not appear to be a primary driver of cytokine induction. Notably, reference strain *E. gallinarum* did not induce inflammatory cytokine expression in the tested biliary and hepatic human cancer cell lines but has been shown to induce inflammatory cytokines in murine hepatic cells derived from mice with a genetic predisposition to lupus-like autoimmunity (29). This result highlights the importance of cell type selection for modeling disease.

Our results identify TNF⍺ and IL-8 as the primary cytokines induced by the PSC-associated bacteria (Fig. 4). However, the other cytokines measured (IL-6, IL-17A, and IFNγ) have all been previously identified as elevated in PSC patients (52, 53). A limitation of our cell assays is that only epithelial cells are included, not immune cells. It is likely that the presence of immune cells would result in the induction of more inflammatory cytokines by the PSC-associated bacteria. Nevertheless, our epithelial cell assays suggest that TNF⍺ and IL-8 may be contributing to biliary inflammation at the local level. These cytokines have been previously shown to be elevated in PSC patient serum, with IL-8 also being significantly elevated in bile, consistent with our results (54).

We observed that *S. salivarius*_PSC01_col01, *E. faecalis*_PSC06_col01, and *V. parvula*_PSC07_col05 upregulate the expression of selected genes involved in sulfur metabolism, mucin expression, and the bicarbonate umbrella, perhaps in response to toxic factors produced by these bacteria. Notably, the bacteria affecting these protective pathways are not the same as the bacteria shown to induce inflammatory cytokines, with the exception of *V. parvula*_PSC07_col05. This finding again supports the notion that bacteria cultured from PSC patient bile affect different aspects of cellular phenotypes, with the exception of *V. parvula*_PSC07_col05*,* which appears to have a mild effect on all observed cellular responses. The two bacterial isolates that consistently produced H_2_S *in vitro* (*E. coli*_PSC10_col01 and *F. necrophorum*_PSC08_col02) did not affect the expression of sulfur metabolism genes in host cells in these targeted analyses (Fig. 5; Table 2). Thus, our results do not suggest that bacterial H_2_S production is driving expression of target sulfur metabolism genes. However, of potential relevance was our observation that supernatants from *V. dispar*_PSC07_col14 cultures caused a decrease in mRNA expression of sulfide quinone oxidoreductase (SQOR) in biliary cells, which could lead to an impairment in H_2_S metabolism. Future work exploring the transcriptional and protein-level effects of H_2_S-producing bacteria on sulfur metabolism in host cells over time is necessary to uncover potential interactions between H_2_S production and host cell damage.

We performed our cellular assays under anaerobic conditions to preserve labile bacterial factors that may degrade or become inactivated upon oxygen exposure. Additionally, these anaerobic 2D cell assays and transwell assays model the low oxygen environment seen at the apical epithelial interface that interacts with anaerobic bacteria, as well as the hypoxic environment associated with fibrotic diseases (19, 20, 55–57). However, we recognize that biliary, liver, and intestinal epithelial cells are vascularized and rely on aerobic metabolism. Although we did not observe hypoxia-induced stress in our anaerobic cell culture experiments, future studies that make use of 3D cell culture models under an oxygen gradient will be important for fully disentangling microbial from hypoxia-driven effects, while maintaining the oxygen-limited environment needed to observe the physiologically relevant effects of anaerobic bacteria. Such 3D cell assays have been successfully used to model intestinal and airway epithelial cell interactions with anaerobic bacteria (55, 57).

**Fig 6:**
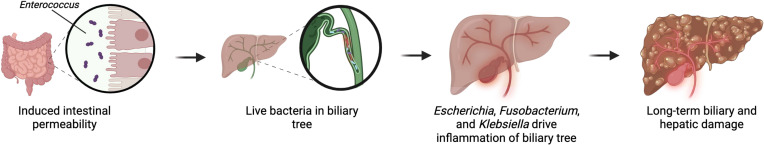
A potential model for microbial involvement in PSC pathogenesis.

Overall, through the culturing of patient-derived bacterial isolates, the collection of sterile-filtered bacterial supernatants, and the use of human cellular assays under anaerobic conditions, we have demonstrated that factors produced by individual PSC-derived bacterial isolates can induce intestinal permeability, inflammation, and cell death. An important caveat of our work is that the effects of the isolates tested here cannot be assumed to be representative of all strains in that species. It is also currently unknown whether gut isolates of the same species would have a similar or different effect as the biliary isolates tested, or whether biliary isolates sourced from different patients would induce different effects. These questions should be investigated in future work. Nonetheless, these results suggest that there may be a causal link between the presence of bacteria in PSC patient microbiomes and disease development. Our work suggests a potential model for PSC pathogenesis in which increased intestinal permeability caused by *Enterococcus* or factors associated with co-occurring IBD may allow bacteria to translocate from the gut to the liver, where these microbes can associate with the biliary tree, either transiently or through colonization. Translocated bacteria such as *Escherichia*, *Fusobacterium*, and *Klebsiella* may then drive inflammation in bile ducts. This inflammation, along with the prolonged exposure to cytotoxic factors produced by PSC-associated bacteria, may then cause or contribute to the progression of biliary fibrosis and hepatic cirrhosis (Fig. 6). Additionally, this model highlights the potential importance of the gut-liver axis in PSC (2), where increased intestinal permeability may allow for both chronic disease and recurrence after liver transplant. We should note that while we propose bacterial translocation from the gut, we cannot rule out other pathways by which bacteria could be transported to the biliary tree, including hematogenous spread of bacteria from the oral microbiome. Finally, bacterial factors may contribute centrally to these critical steps in the pathogenesis of PSC, but likely operate in conjunction with other genetic, environmental, or autoimmune contributions to intestinal permeability, inflammation, and cell death.

The connections presented in this study between specific bacteria and cellular responses open up new avenues for identifying the underlying causes of PSC. While our use of bacterial supernatants and biological replicates has allowed us to demonstrate an initial link between strains and cellular responses, future studies are needed to identify what specific factors, such as proteins or metabolites, are being produced by these bacteria and what quantity of these factors is needed to induce disease. This further examination could potentially allow for the identification of novel therapeutic targets for PSC.

## MATERIALS AND METHODS

### Bile collection

Bile was collected during ERCPs being performed for clinical indications. Collection methods were designed to minimize contamination from the oral cavity and gastrointestinal tract. Once the scope was introduced into the duodenum, the channel was flushed with 20 cc of sterile saline. The catheter was then inserted into the scope and flushed with 5 cc of sterile saline. The catheter was introduced into the biliary tree, and at least 3 cc of bile was withdrawn into a sterile empty syringe. The syringe was immediately capped. A second syringe of bile was then drawn until full and immediately capped to limit the amount of headspace in the syringe and minimize potential gas exchange during transport. The first syringe of bile was stored for biobanking, and the second syringe was used for bacterial culturing. The sample was not placed on ice or dry ice or otherwise stored, but was immediately transported for culturing. To serve as controls, non-PSC bile was collected during cholecystectomies. Informed consent was obtained. The control bile was obtained soon after cholecystectomy in the operating room. Patients were selected who did not have any active inflammatory disease or cholecystitis and had not been on an antibiotic for at least 4 weeks. A needle was used to pierce through the gallbladder wall, and a syringe was used to withdraw at least 3 cc of bile. The syringe was then capped and brought immediately to the lab.

### Bacterial culturing and isolate generation

On the same day of patient bile collection, bacterial culturing was performed in an anaerobic chamber (Coy Laboratory Products) with a gas mixture of 5% H_2_ and 20% CO_2_ (balance N_2_). Pure bile (100 µL) or bile dilutions in BHI+ medium (brain heart infusion [BHI, Bacto] supplemented with 1% BBL vitamin K1-hemin solution [BD], 1% trace minerals solution [ATCC], 1% trace vitamins [ATCC], 5% heat-inactivated fetal bovine serum [FBS, Hyclone], 1 g/L cellobiose, 1 g/L maltose, 1 g/L fructose, and 0.5 g/L L-cysteine) were plated on BHI+ agar plates. BHI is a rich medium that supports the growth of a diverse array of anaerobic bacteria (58), and the additives further support the growth of fastidious microorganisms from the mammalian gut (41–43). Dilutions were made at 10^−1^, 10^−2^, 10^−3^, and 10^−4^. Plates were incubated at 37°C for 2–3 days. Individual colonies (50 per sample, prioritizing the collection of diverse sizes and colony morphologies whenever possible) were then picked and streaked onto BHI+ plates for another 2–3 day incubation at 37°C. When we were unable to detect growth after 2–3 days, bacterial liquid cultures and agar plates were left to incubate for 1 week. In these cases, no growth was observed, and the plates were determined to be sterile. To ensure isolate purity, the 50 streaked colonies were picked and streaked on BHI+ plates a second time. After incubation, single colonies from re-streaked agar plates were inoculated into 1 mL of BHI+ medium and incubated at 37°C for 3 days. An aliquot (10 µL) of each culture was inoculated into 1 mL of SIM+ medium (BBL SIM medium [BD] supplemented with 1% BBL vitamin K1-hemin solution [BD] and 0.5 g/L L-cysteine) to identify colonies that produced hydrogen sulfide (H_2_S), using *Escherichia coli* Nissle 1917 as a positive control (originally obtained from the Silver laboratory at Harvard Medical School). Another aliquot (500 µL) of the isolate BHI+ cultures was used for identification by 16S rRNA gene PCR and sequencing (see below), while the remaining isolate cultures were stored at −80°C as glycerol stocks.

### 16S rRNA gene sequencing (Sanger method) and clonality analysis

An aliquot (500 µL) of bacterial culture was centrifuged for 2 minutes at maximum speed, and the supernatant was discarded. The pellet was resuspended in 100 µL Prepman Ultra sample prep reagent (Thermo Fisher), transferred to PCR tubes, and incubated at 100°C for 10 minutes in a thermocycler to lyse the cells. The tubes were briefly centrifuged to remove cell debris, and the supernatant was saved and transferred to a new tube. PCR amplification was performed using universal 16S primers (universal 16S-forward, 5′-GAGTTTGATCCTGGCTCAG-3′; universal 16S-reverse, 5′-GGCTACCTTGTTACGACTT-3′). The product was treated with ExoSAP (Thermo Fisher) to remove excess primer and dNTPs.

16S rRNA gene sequencing (Sanger method) was performed by Genewiz using the universal 16S-reverse primer 5′-GGCTACCTTGTTACGACTT-3′. Raw, untrimmed sequences were used to hypothesize species identity. Taxonomic assignment used NCBI BLASTn against NCBI nt. Species name(s) with the lowest e-values and highest percent identities and query coverage were recorded (see Tables S2 to S10). Sequence quality was determined by Genewiz.

To assess isolate clonality, sequences were trimmed for quality using Galaxy "trim sequences" tool to 800 bp by removing low-quality leading and trailing regions to standardize all sequences to the same core 16S rRNA gene region (59). Where 10 or more colonies from the same donor were identified as the same species, the trimmed 16S sequences with the same species designation were aligned to each other using the ClustalOmega (60) plugin in Geneious Prime analysis software. These alignments were trimmed to a region that was covered by all sequences (~700 bp always encompassing V5–V7). Trimmed alignments were then manually curated to designate clonal groups within a species. Clonal groups were designated when there was a set of isolates with the same nucleotide sequence (100% identity) across the shared overlap. These groups are indicated in Tables S2 to S10. Clonal group numbers within a species were assigned based on how many isolates were in a group (i.e., group 1 has the most isolates). The numbering order for equally represented groups is arbitrary. Entries are marked “N/A” when there are fewer than 10 isolates for a given species, when there was poor read quality, or when there was only genus-level identification.

### gDNA extraction from bile and metagenomic sequencing

Genomic DNA (gDNA) was isolated from bile using the PureLink Genomic DNA Mini Kit (Invitrogen) following the manufacturer’s instructions for extraction from blood samples. Samples requiring concentration for sequencing were prepared by SeqCenter (Pittsburgh, PA) using AMPure XP Bead-based reagent. Libraries were prepared by SeqCenter using the Illumina DNA Prep Kit (Illumina) and IDT 10 bp UDI indices and sequenced on an Illumina NovaSeq X Plus sequencer, producing 2 × 151 bp paired-end reads resulting in 5 Gbp of data per sample (32M reads). Quality filtering, including demultiplexing and adapter trimming, was performed by SeqCenter using bcl-convert (v4.2.4, Illumina). Taxonomic abundances were analyzed using MetaPhlan 4.0 (61), and figures were generated using GraphPad Prism 10. Sequencing statistics, including the total number of reads, are reported in Table S13. Metagenomic data have been deposited in the NCBI BioProject database (PRJNA1169933) and are publicly available. Accession numbers are listed in Table S14. Attempted isolation of gDNA from bile using the QIAmp DNA Microbiome Kit (Qiagen) or the PowerSoil Pro Extraction Kit (Qiagen), with or without concentration and purification by the PowerClean Pro Kit (Qiagen), did not result in enough gDNA of sufficient purity for metagenomic sequencing. Bile pH was not predictive of extracted gDNA quantity or purity (data not shown).

### Human cell culture

Caco-2 and HepG2 cells were purchased from ATCC, while EGI-1 cells were purchased from DSMZ. Cells were cultured in Dulbecco’s modified Eagle medium (DMEM) containing glucose and L-glutamine (Gibco), supplemented with 10% FBS (Genclone) and 1% penicillin/streptomycin. Mycoplasma testing was performed using a MycoAlert Mycoplasma Detection Kit (Lonza), and all lines were negative.

### Bacterial supernatant collection for cellular assays

Bacterial cultures were normalized to an OD_600_ of 0.1 in BHI+ medium and grown anaerobically at 37°C overnight before the day of human cell assays. Final OD_600_ values were measured to be in the range of their respective observed stationary phases (Table S15) prior to supernatant collection for all human cell experiments. However, these isolates have different growth behaviors and may likely have different carrying capacities in BHI+ medium, which was not accounted for in our methods. After incubation, bacterial supernatants were collected inside the anaerobic chamber by sterile filtration using a 0.2 µm Supor Membrane syringe filter (Pall Corporation) to maintain any bacterially produced gases as previously described (62). Collected supernatants were immediately used in cell assays. *Enterococcus gallinarum* (gift from Martin Kriegel at Yale School of Medicine) (29) and *Bacteroides fragilis* (ATCC 25285) were used as reference strains and were not derived from PSC patients.

### Cell viability

The day before treatment, 30,000 Caco-2, HepG2, or EGI-1 cells were plated in 100 µL of complete DMEM in a 96-well white Nunclon Delta-treated plate (ThermoFisher) and incubated at 37°C with 5% CO_2_ overnight. The day of the assay, the medium was aspirated, and the cells were moved into the anaerobic chamber. Anaerobic complete DMEM was added to the wells and cells were incubated at 37°C for 1 h before adding sterile-filtered bacterial supernatants such that the final well volumes were 100 µL of supernatant and DMEM at ratios of 1:1 or 1:3 (vol/vol). Cells were then incubated anaerobically at 37°C for 16 h before viability was assessed using the CellTiter-Glo luminescent cell viability assay (Promega), following the manufacturer’s standards. Cells maintained standard appearance and expected adhesion and a high signal-to-noise ratio for the assay (2500, 2200, and 2900 for EGI-1, Caco-2, and HepG2 cells, respectively), indicating minimal cell stress due to anoxia alone. The luminescence signal was normalized to wells with supernatant and DMEM without human cells.

### Epithelial permeability assay

Caco-2 cells were differentiated in 24-well plate transwells (0.4 µM pore size, Costar) as previously described (33). On the day of the assay, differentiated Caco-2 cells were washed with Hank’s balanced salt solution (HBSS, Gibco) before being moved into the anaerobic chamber. Anaerobic HBSS (500 µL) was added to the basal chamber, and 50 µL of anaerobic HBSS with 10 µM 4 kDa FITC-Dextran (Sigma-Aldrich) was added to each apical chamber before incubation at 37°C for 1 h. Syringe-filtered bacterial supernatants (50 µL) were then added to each well, and the cells were incubated at 37°C for 6 or 12 h. HBSS (100 µL) from the basal chamber was then moved to a black 96-well plate (Corning), and fluorescence was measured using an Envision 2104 plate reader at Harvard Medical School’s ICCB-Longwood Screening Facility.

### Gene expression analysis by qRT-PCR

The day before treatment, 1,000,000 EGI-1 or HepG2 cells were plated in 2 mL complete DMEM in six-well Nunclon Delta-treated plates (ThermoFisher) and incubated at 37°C with 5% CO_2_ overnight. The day of the assay, the medium was aspirated, and the cells were moved into the anaerobic chamber. Anaerobic complete DMEM (1 mL) was added to the wells, and the cells were incubated at 37°C for 1 h. Next, sterile-filtered bacterial supernatant (1 mL) was added to each well and cells were incubated at 37°C for 6 h. Cells were then removed from the anaerobic chamber, the medium was aspirated, and the cells were washed with 1 mL ice-cold PBS. Cells were lysed in ice-cold TRI Reagent (Zymo Research) and stored at −80°C until RNA was extracted using the Direct-zol RNA Miniprep Plus kit (Zymo Research) with the DNase cleanup step. RNA (1 µg per sample) was reverse transcribed using the High-Capacity cDNA Reverse Transcription kit (Applied Biosystems). cDNA (10 ng per sample) was then analyzed by qRT-PCR using LightCycler 480 SYBR Green I Master (Roche). Reactions were performed in a 384-well format on a QuantStudio 7 Pro at Harvard Medical School’s ICCB-Longwood Screening Facility. The 2^−ΔΔCt^ method (63) was used to calculate relative changes in gene expression, and all results were normalized to UBC mRNA expression. Primer sequences were obtained from PrimerBank (https://pga.mgh.harvard.edu/primerbank/). Primer efficiencies were determined to be between 90% and 110% with R^2^ values between 0.99 and 1.00. Sequences are provided in the supplemental material (Table S16).

## Data Availability

We will make data fully available and without restriction. The NCBI BioProject number for the metagenomic data is PRJNA1169933. Accession numbers for each sample are available in Table S14.

## SUPPLEMENTAL MATERIAL

Supplemental Information msphere.00550-25-s0001.pdf Figure S1 and Tables S1-S16.

ASM does not own the copyrights to Supplemental Material that may be linked to, or accessed through, an article. The authors have granted ASM a non-exclusive, world-wide license to publish the Supplemental Material files. Please contact the corresponding author directly for reuse.

## References

[1] Karlsen TH, Folseraas T, Thorburn D, Vesterhus M. 2017. Primary sclerosing cholangitis - a comprehensive review. J Hepatol 67:1298–1323. doi:10.1016/j.jhep.2017.07.022 28802875

[2] Hov JR, Karlsen TH. 2023. The microbiota and the gut–liver axis in primary sclerosing cholangitis. Nat Rev Gastroenterol Hepatol 20:135–154. doi:10.1038/s41575-022-00690-y 36352157

[3] Sayed A, Silveira M, Assis D, Deng Y, Gaidos J, Proctor D, Al-Bawardy B. 2022. Predictors and outcomes of biologic/immunosuppressive therapy for psc-associated IBD. Gastroenterology 162:S76. doi:10.1053/j.gastro.2021.12.161

[4] Vesterhus M, Karlsen TH. 2020. Emerging therapies in primary sclerosing cholangitis: pathophysiological basis and clinical opportunities. J Gastroenterol 55:588–614. doi:10.1007/s00535-020-01681-z 32222826 PMC7242240

[5] Fosby B, Karlsen TH, Melum E. 2012. Recurrence and rejection in liver transplantation for primary sclerosing cholangitis. World J Gastroenterol 18:1–15. doi:10.3748/wjg.v18.i1.1 22228965 PMC3251800

[6] Jiang X, Karlsen TH. 2017. Genetics of primary sclerosing cholangitis and pathophysiological implications. Nat Rev Gastroenterol Hepatol 14:279–295. doi:10.1038/nrgastro.2016.154 28293027

[7] Hov JR, Kummen M. 2017. Intestinal microbiota in primary sclerosing cholangitis. Curr Opin Gastroenterol 33:85–92. doi:10.1097/MOG.0000000000000334 28030369

[8] Bajer L, Kverka M, Kostovcik M, Macinga P, Dvorak J, Stehlikova Z, Brezina J, Wohl P, Spicak J, Drastich P. 2017. Distinct gut microbiota profiles in patients with primary sclerosing cholangitis and ulcerative colitis. World J Gastroenterol 23:4548–4558. doi:10.3748/wjg.v23.i25.4548 28740343 PMC5504370

[9] Kummen M, Thingholm LB, Rühlemann MC, Holm K, Hansen SH, Moitinho-Silva L, Liwinski T, Zenouzi R, Storm-Larsen C, Midttun Ø, McCann A, Ueland PM, Høivik ML, Vesterhus M, Trøseid M, Laudes M, Lieb W, Karlsen TH, Bang C, Schramm C, Franke A, Hov JR. 2021. Altered gut microbial metabolism of essential nutrients in primary sclerosing cholangitis. Gastroenterology 160:1784–1798. doi:10.1053/j.gastro.2020.12.058 33387530 PMC7611822

[10] Liu Q, Li B, Li Y, Wei Y, Huang B, Liang J, You Z, Li Y, Qian Q, Wang R, Zhang J, Chen R, Lyu Z, Chen Y, Shi M, Xiao X, Wang Q, Miao Q, Fang J-Y, Gershwin ME, Lian M, Ma X, Tang R. 2022. Altered faecal microbiome and metabolome in IgG4-related sclerosing cholangitis and primary sclerosing cholangitis. Gut 71:899–909. doi:10.1136/gutjnl-2020-323565 34035120

[11] Kummen M, Holm K, Anmarkrud JA, Nygård S, Vesterhus M, Høivik ML, Trøseid M, Marschall H-U, Schrumpf E, Moum B, Røsjø H, Aukrust P, Karlsen TH, Hov JR. 2017. The gut microbial profile in patients with primary sclerosing cholangitis is distinct from patients with ulcerative colitis without biliary disease and healthy controls. Gut 66:611–619. doi:10.1136/gutjnl-2015-310500 26887816

[12] Liwinski T, Zenouzi R, John C, Ehlken H, Rühlemann MC, Bang C, Groth S, Lieb W, Kantowski M, Andersen N, Schachschal G, Karlsen TH, Hov JR, Rösch T, Lohse AW, Heeren J, Franke A, Schramm C. 2020. Alterations of the bile microbiome in primary sclerosing cholangitis. Gut 69:665–672. doi:10.1136/gutjnl-2019-318416 31243055

[13] Pereira P, Aho V, Arola J, Boyd S, Jokelainen K, Paulin L, Auvinen P, Färkkilä M. 2017. Bile microbiota in primary sclerosing cholangitis: impact on disease progression and development of biliary dysplasia. PLoS One 12:e0182924. doi:10.1371/journal.pone.0182924 28796833 PMC5552186

[14] Forster K, Klein F, Simon N, Chhatwal P, Ziesing S, Heidrich B, Solbach P. 2023. Cultivation-based characterization of biliary microbiota in bile samples collected during routine endoscopic retrograde cholangiography: 10 years of experience at a tertiary center. Eur J Gastroenterol Hepatol 35:1159–1167. doi:10.1097/MEG.0000000000002633 37577778

[15] D’Amico F, Bertacco A, Finotti M, Di Renzo C, Rodriguez-Davalos MI, Gondolesi GE, Cillo U, Mulligan D, Geibel J. 2021. Bile microbiota in liver transplantation: proof of concept using gene amplification in a heterogeneous clinical scenario. Front Surg 8:621525. doi:10.3389/fsurg.2021.621525 33796547 PMC8009296

[16] Molinero N, Ruiz L, Milani C, Gutiérrez-Díaz I, Sánchez B, Mangifesta M, Segura J, Cambero I, Campelo AB, García-Bernardo CM, Cabrera A, Rodríguez JI, González S, Rodríguez JM, Ventura M, Delgado S, Margolles A. 2019. The human gallbladder microbiome is related to the physiological state and the biliary metabolic profile. Microbiome 7:100. doi:10.1186/s40168-019-0712-8 31272480 PMC6610825

[17] Nakamoto N, Sasaki N, Aoki R, Miyamoto K, Suda W, Teratani T, Suzuki T, Koda Y, Chu P-S, Taniki N, Yamaguchi A, Kanamori M, Kamada N, Hattori M, Ashida H, Sakamoto M, Atarashi K, Narushima S, Yoshimura A, Honda K, Sato T, Kanai T. 2019. Gut pathobionts underlie intestinal barrier dysfunction and liver T helper 17 cell immune response in primary sclerosing cholangitis. Nat Microbiol 4:492–503. doi:10.1038/s41564-018-0333-1 30643240

[18] Ichikawa M, Nakamoto N, Kredo-Russo S, Weinstock E, Weiner IN, Khabra E, Ben-Ishai N, Inbar D, Kowalsman N, Mordoch R, et al.. 2023. Bacteriophage therapy against pathological Klebsiella pneumoniae ameliorates the course of primary sclerosing cholangitis. Nat Commun 14:3261. doi:10.1038/s41467-023-39029-9 37277351 PMC10241881

[19] Cai J, Hu M, Chen Z, Ling Z. 2021. The roles and mechanisms of hypoxia in liver fibrosis. J Transl Med 19:186. doi:10.1186/s12967-021-02854-x 33933107 PMC8088569

[20] Zhao J, Yue P, Mi N, Li M, Fu W, Zhang X, Gao L, Bai M, Tian L, Jiang N, et al.. 2024. Biliary fibrosis is an important but neglected pathological feature in hepatobiliary disorders: from molecular mechanisms to clinical implications. Medical Review 4:326–365. doi:10.1515/mr-2024-0029 39135601 PMC11317084

[21] Yin X, Sasson G, Sun Z, Ke S, Babazadeh D, Mahmood SD, Andrews M, Hurwitz S, Chikowore T, Paul M, Javier N, Dave M, Austin A, Gray L, Steinberg F, Souza E, Bowlus C, Liu Y-Y, Korzenik J. 2024. Demonstrating the beneficial effect of low protein diet in primary sclerosing cholangitis through a randomized clinical trial and multi-omics data analysis. Gastroenterology. doi:10.1101/2024.02.23.24303167

[22] Stummer N, Feichtinger RG, Weghuber D, Kofler B, Schneider AM. 2023. Role of hydrogen sulfide in inflammatory bowel disease. Antioxidants (Basel) 12:1570. doi:10.3390/antiox12081570 37627565 PMC10452036

[23] Teigen LM, Geng Z, Sadowsky MJ, Vaughn BP, Hamilton MJ, Khoruts A. 2019. Dietary factors in sulfur metabolism and pathogenesis of ulcerative colitis. Nutrients 11:931. doi:10.3390/nu11040931 31027194 PMC6521024

[24] Rowan FE, Docherty NG, Coffey JC, O’Connell PR. 2009. Sulphate-reducing bacteria and hydrogen sulphide in the aetiology of ulcerative colitis. Br J Surg 96:151–158. doi:10.1002/bjs.6454 19160346

[25] Khalil NA, Walton GE, Gibson GR, Tuohy KM, Andrews SC. 2014. In vitro batch cultures of gut microbiota from healthy and ulcerative colitis (UC) subjects suggest that sulphate-reducing bacteria levels are raised in UC and by a protein-rich diet. Int J Food Sci Nutr 65:79–88. doi:10.3109/09637486.2013.825700 23941288

[26] Iwasawa K, Suda W, Tsunoda T, Oikawa-Kawamoto M, Umetsu S, Inui A, Fujisawa T, Morita H, Sogo T, Hattori M. 2017. Characterisation of the faecal microbiota in Japanese patients with paediatric-onset primary sclerosing cholangitis. Gut 66:1344–1346. doi:10.1136/gutjnl-2016-312533 27670376 PMC5530472

[27] Sabino J, Vieira-Silva S, Machiels K, Joossens M, Falony G, Ballet V, Ferrante M, Van Assche G, Van der Merwe S, Vermeire S, Raes J. 2016. Primary sclerosing cholangitis is characterised by intestinal dysbiosis independent from IBD. Gut 65:1681–1689. doi:10.1136/gutjnl-2015-311004 27207975 PMC5036217

[28] He Q, Niu M, Bi J, Du N, Liu S, Yang K, Li H, Yao J, Du Y, Duan Y. 2023. Protective effects of a new generation of probiotic bacteroides fragilis against colitis in vivo and in vitro*.* Sci Rep 13:15842. doi:10.1038/s41598-023-42481-8 37740010 PMC10517118

[29] Manfredo Vieira S, Hiltensperger M, Kumar V, Zegarra-Ruiz D, Dehner C, Khan N, Costa FRC, Tiniakou E, Greiling T, Ruff W, Barbieri A, Kriegel C, Mehta SS, Knight JR, Jain D, Goodman AL, Kriegel MA. 2018. Translocation of a gut pathobiont drives autoimmunity in mice and humans. Science 359:1156–1161. doi:10.1126/science.aar7201 29590047 PMC5959731

[30] Wang Y, Deng H, Li Z, Tan Y, Han Y, Wang X, Du Z, Liu Y, Yang R, Bai Y, Bi Y, Zhi F. 2017. Safety evaluation of a novel strain of bacteroides fragilis. Front Microbiol 8:435. doi:10.3389/fmicb.2017.00435 28367145 PMC5355466

[31] Rabizadeh S, Rhee K-J, Wu S, Huso D, Gan CM, Golub JE, Wu X, Zhang M, Sears CL. 2007. Enterotoxigenic bacteroides fragilis: a potential instigator of colitis. Inflamm Bowel Dis 13:1475–1483. doi:10.1002/ibd.20265 17886290 PMC3056612

[32] Zamani S, Hesam Shariati S, Zali MR, Asadzadeh Aghdaei H, Sarabi Asiabar A, Bokaie S, Nomanpour B, Sechi LA, Feizabadi MM. 2017. Detection of enterotoxigenic Bacteroides fragilis in patients with ulcerative colitis. Gut Pathog 9:53. doi:10.1186/s13099-017-0202-0 28924454 PMC5599888

[33] Chaudhari S, Devlin A. 2021. Intestinal co-culture system to study TGR5 agonism and gut restriction. Bio Protoc 11:BIO–Protoc doi:10.21769/BioProtoc.3948 PMC8032489 33855108

[34] Lea T. 2015. The Impact of Food Bioactives on Health, in vitro and ex vivo models, p 103–111 29787039

[35] Deng H, Yang S, Zhang Y, Qian K, Zhang Z, Liu Y, Wang Y, Bai Y, Fan H, Zhao X, Zhi F. 2018. Bacteroides fragilis prevents Clostridium difficile infection in a mouse model by restoring gut barrier and microbiome regulation. Front Microbiol 9:2976. doi:10.3389/fmicb.2018.02976 30619112 PMC6308121

[36] Carpino G, Cardinale V, Renzi A, Hov JR, Berloco PB, Rossi M, Karlsen TH, Alvaro D, Gaudio E. 2015. Activation of biliary tree stem cells within peribiliary glands in primary sclerosing cholangitis. J Hepatol 63:1220–1228. doi:10.1016/j.jhep.2015.06.018 26119688

[37] Beatty P, Yan I, Francis F, Nakhleh R, Harnois D, Farraye FA, Patel T, Hashash JG. 2023. S1508 Patterns of abnormal MUC1 expression in patients with PSC and cholangiocarcinoma. Am J Gastroenterol 118:S1143–S1144. doi:10.14309/01.ajg.0000955672.03480.07

[38] Rupp C, Bode KA, Leopold Y, Sauer P, Gotthardt DN. 2018. Pathological features of primary sclerosing cholangitis identified by bile proteomic analysis. Biochimica et Biophysica Acta (BBA) - Molecular Basis of Disease 1864:1380–1389. doi:10.1016/j.bbadis.2017.09.012 28943450

[39] Dyson JK, Beuers U, Jones DEJ, Lohse AW, Hudson M. 2018. Primary sclerosing cholangitis. Lancet 391:2547–2559. doi:10.1016/S0140-6736(18)30300-3 29452711

[40] Watanabe S, Minagawa M, Shinoda T, Motooka D, Tohya M, Kirikae T, Nakamura S, Saiura A. 2022. Bile collected from the normal gallbladder of patients during surgery has simple bacterial flora. Cureus 14:e25681. doi:10.7759/cureus.25681 35812645 PMC9257430

[41] Hall AB, Yassour M, Sauk J, Garner A, Jiang X, Arthur T, Lagoudas GK, Vatanen T, Fornelos N, Wilson R, Bertha M, Cohen M, Garber J, Khalili H, Gevers D, Ananthakrishnan AN, Kugathasan S, Lander ES, Blainey P, Vlamakis H, Xavier RJ, Huttenhower C. 2017. A novel Ruminococcus gnavus clade enriched in inflammatory bowel disease patients. Genome Med 9:103. doi:10.1186/s13073-017-0490-5 29183332 PMC5704459

[42] Chen Y, Chaudhari SN, Harris DA, Roberts CF, Moscalu A, Mathur V, Zhao L, Tavakkoli A, Devlin AS, Sheu EG. 2024. A small intestinal bile acid modulates the gut microbiome to improve host metabolic phenotypes following bariatric surgery. Cell Host Microbe 32:1315–1330. doi:10.1016/j.chom.2024.06.014 39043190 PMC11332993

[43] Paik D, Yao L, Zhang Y, Bae S, D’Agostino GD, Zhang M, Kim E, Franzosa EA, Avila-Pacheco J, Bisanz JE, Rakowski CK, Vlamakis H, Xavier RJ, Turnbaugh PJ, Longman RS, Krout MR, Clish CB, Rastinejad F, Huttenhower C, Huh JR, Devlin AS. 2022. Human gut bacteria produce ΤΗ17-modulating bile acid metabolites. Nature 603:907–912. doi:10.1038/s41586-022-04480-z 35296854 PMC9132548

[44] Lu J, Rincon N, Wood DE, Breitwieser FP, Pockrandt C, Langmead B, Salzberg SL, Steinegger M. 2022. Metagenome analysis using the Kraken software suite. Nat Protoc 17:2815–2839. doi:10.1038/s41596-022-00738-y 36171387 PMC9725748

[45] Ondov BD, Bergman NH, Phillippy AM. 2011. Interactive metagenomic visualization in a web browser. BMC Bioinformatics 12:385. doi:10.1186/1471-2105-12-385 21961884 PMC3190407

[46] Pomyen Y, Chaisaingmongkol J, Rabibhadana S, Pupacdi B, Sripan D, Chornkrathok C, Budhu A, Budhisawasdi V, Lertprasertsuke N, Chotirosniramit A, Pairojkul C, Auewarakul CU, Ungtrakul T, Sricharunrat T, Phornphutkul K, Sangrajang S, Loffredo CA, Harris CC, Mahidol C, Wang XW, Ruchirawat M, TIGER-LC Consortium. 2023. Gut dysbiosis in thai intrahepatic cholangiocarcinoma and hepatocellular carcinoma. Sci Rep 13:11406. doi:10.1038/s41598-023-38307-2 37452065 PMC10349051

[47] Troy EB, Kasper DL. 2010. Beneficial effects of bacteroides fragilis polysaccharides on the immune system. Front Biosci 15:25. doi:10.2741/3603 PMC2995369 20036803

[48] Hsiao EY, McBride SW, Hsien S, Sharon G, Hyde ER, McCue T, Codelli JA, Chow J, Reisman SE, Petrosino JF, Patterson PH, Mazmanian SK. 2013. Microbiota modulate behavioral and physiological abnormalities associated with neurodevelopmental disorders. Cell 155:1451–1463. doi:10.1016/j.cell.2013.11.024 24315484 PMC3897394

[49] Maharshak N, Huh EY, Paiboonrungruang C, Shanahan M, Thurlow L, Herzog J, Djukic Z, Orlando R, Pawlinski R, Ellermann M, Borst L, Patel S, Dotan I, Sartor RB, Carroll IM. 2015. Enterococcus faecalis gelatinase mediates intestinal permeability via protease-activated receptor 2. Infect Immun 83:2762–2770. doi:10.1128/IAI.00425-15 25916983 PMC4468563

[50] Qin J, Li R, Raes J, Arumugam M, Burgdorf KS, Manichanh C, Nielsen T, Pons N, Levenez F, Yamada T, et al.. 2010. A human gut microbial gene catalogue established by metagenomic sequencing. Nature 464:59–65. doi:10.1038/nature08821 20203603 PMC3779803

[51] Próchnicki T, Vasconcelos MB, Robinson KS, Mangan MSJ, De Graaf D, Shkarina K, Lovotti M, Standke L, Kaiser R, Stahl R, Duthie FG, Rothe M, Antonova K, Jenster L-M, Lau ZH, Rösing S, Mirza N, Gottschild C, Wachten D, Günther C, Kufer TA, Schmidt FI, Zhong FL, Latz E. 2023. Mitochondrial damage activates the NLRP10 inflammasome. Nat Immunol 24:595–603. doi:10.1038/s41590-023-01451-y 36941400

[52] Dold L, Frank L, Lutz P, Kaczmarek DJ, Krämer B, Nattermann J, Weismüller TJ, Branchi V, Toma M, Gonzalez-Carmona M, Strassburg CP, Spengler U, Langhans B. 2023. IL-6-dependent STAT3 activation and induction of proinflammatory cytokines in primary sclerosing cholangitis. Clin Transl Gastroenterol 14:e00603. doi:10.14309/ctg.0000000000000603 37256725 PMC10461951

[53] Moreno ASG, Guicciardi ME, Wixom AQ, Jessen E, Yang J, Ilyas SI, Bianchi JK, Vairo FP e, Lazaridis KN, Gores GJ. 2024. IL-17 signaling in primary sclerosing cholangitis patient-derived organoids. Hepatol Commun. doi:10.21203/rs.3.rs-3406046/v1 PMC11150034 38829197

[54] Zweers SJ, Shiryaev A, Komuta M, Vesterhus M, Hov JR, Perugorria MJ, de Waart DR, Chang J-C, Tol S, Te Velde AA, de Jonge WJ, Banales JM, Roskams T, Beuers U, Karlsen TH, Jansen PL, Schaap FG. 2016. Elevated interleukin-8 in bile of patients with primary sclerosing cholangitis. Liver Int 36:1370–1377. doi:10.1111/liv.13092 26866350

[55] Jalili-Firoozinezhad S, Gazzaniga FS, Calamari EL, Camacho DM, Fadel CW, Bein A, Swenor B, Nestor B, Cronce MJ, Tovaglieri A, Levy O, Gregory KE, Breault DT, Cabral JMS, Kasper DL, Novak R, Ingber DE. 2019. A complex human gut microbiome cultured in an anaerobic intestine-on-a-chip. Nat Biomed Eng 3:520–531. doi:10.1038/s41551-019-0397-0 31086325 PMC6658209

[56] Guo R, Xu X, Lu Y, Xie X. 2017. Physiological oxygen tension reduces hepatocyte dedifferentiation in in vitro culture. Sci Rep 7:5923. doi:10.1038/s41598-017-06433-3 28724942 PMC5517567

[57] Moore PJ, Hoffman K, Ahmed S, Fletcher JR, Wiggen TD, Lucas SK, Arif SJ, Gilbertsen AJ, Kent LA, Fiege JK, Langlois RA, O’Grady SM, Hunter RC. 2025. Dual oxic-anoxic co-culture enables direct study of anaerobe–host interactions at the airway epithelial interface. mBio 16:e01338–24. doi:10.1128/mbio.01338-24 40366160 PMC12077211

[58] Javdan B, Lopez JG, Chankhamjon P, Lee Y-C, Hull R, Wu Q, Wang X, Chatterjee S, Donia MS. 2020. Personalized mapping of drug metabolism by the human gut microbiome. Cell 181:1661–1679. doi:10.1016/j.cell.2020.05.001 32526207 PMC8591631

[59] Afgan E, Nekrutenko A, Grüning BA, Blankenberg D, Goecks J, Schatz MC, Ostrovsky AE, Mahmoud A, Lonie AJ, Syme A, et al.. 2022. The galaxy platform for accessible, reproducible and collaborative biomedical analyses: 2022 update. Nucleic Acids Res 50:W345–W351. doi:10.1093/nar/gkac247 35446428 PMC9252830

[60] Thompson JD, Higgins DG, Gibson TJ. 1994. CLUSTAL W: improving the sensitivity of progressive multiple sequence alignment through sequence weighting, position-specific gap penalties and weight matrix choice. Nucleic Acids Res 22:4673–4680. doi:10.1093/nar/22.22.4673 7984417 PMC308517

[61] Blanco-Míguez A, Beghini F, Cumbo F, McIver LJ, Thompson KN, Zolfo M, Manghi P, Dubois L, Huang KD, Thomas AM, et al.. 2023. Extending and improving metagenomic taxonomic profiling with uncharacterized species using MetaPhlAn 4. Nat Biotechnol 41:1633–1644. doi:10.1038/s41587-023-01688-w 36823356 PMC10635831

[62] McCurry MD, D’Agostino GD, Walsh JT, Bisanz JE, Zalosnik I, Dong X, Morris DJ, Korzenik JR, Edlow AG, Balskus EP, Turnbaugh PJ, Huh JR, Devlin AS. 2024. Gut bacteria convert glucocorticoids into progestins in the presence of hydrogen gas. Cell 187:2952–2968. doi:10.1016/j.cell.2024.05.005 38795705 PMC11179439

[63] Livak KJ, Schmittgen TD. 2001. Analysis of relative gene expression data using real-time quantitative PCR and the 2^−ΔΔC_T_^ method. Methods 25:402–408. doi:10.1006/meth.2001.1262 11846609

