# Supplementary material for: Cultured bacteria isolated from primary sclerosing cholangitis patient bile induce inflammation and cell death
Source: mSphere. 2025 Oct 20;10(11):e00550-25. doi: 10.1128/msphere.00550-25 (PMC12645923; doi:10.1128/msphere.00550-25)
Supplement: Supplemental Information — Figure S1 and Tables S1-S16. [file msphere.00550-25-s0001.pdf]

Supplemental Information

Cultured bacteria isolated from primary sclerosing cholangitis patient bile induce inflammation and cell death

Chelsea E. Powell,[a] Megan D. McCurry,[a] Silvia Fernanda Quevedo,[b] Lindsay Ventura,[c] Kumar Krishnan,[c] Malav Dave,[b] Shaikh Danish Mahmood,[b] Katherine Specht,[c] Raghav Bordia,[c] Molly R. Sargen,[d] Daniel S. Pratt,[c] Joshua R. Korzenik,[b,#] A. Sloan Devlin[a,#]

[a]*Department of Biological Chemistry and Molecular Pharmacology, Harvard Medical School, Boston, MA, USA*

[b]*Division of Gastroenterology, Hepatology and Endoscopy, Brigham & Women's Hospital, Boston, MA, USA*

[c]*Autoimmune and Cholestatic Liver Center, Massachusetts General Hospital, Boston, MA, USA*

[d]*Department of Microbiology, Harvard Medical School, Boston, MA, USA*

Running Head: Cultured PSC bacteria cause cell damage

[#] Address correspondence to Joshua R. Korzenik (JKORZENIK@bwh.harvard.edu) and A. Sloan Devlin (sloan_devlin@g.harvard.edu)

Keywords: Primary sclerosing cholangitis, bacterial isolates, biliary microbiome, cellular assays, cellular phenotype

Table S1. Individual patient characteristics at time of bile collection

Patient	Age	Sex	PSC Diagnosis	PSC Duration (years)	IBD Diagnosis	IBD Duration (years)	ERCP (#)	ALP (U/L)
PSC 1	38	Male	PSC	0	IC	22	1	58
PSC 2	34	Male	PSC large duct	6	UC/CD	6	3	621
PSC 3	73	Male	PSC	27	UC	50+	17	289
PSC 4	67	Female	PSC	21	None	N/A	7	143
PSC 5	32	Male	PSC	8	CD	3	4	629
PSC 6	38	Male	PSC	9	UC	14	4	136
PSC 7	28	Male	PSC	5	None	N/A	1	554
PSC 8	58	Male	PSC	10	UC	12	9	472
PSC 9	73	Male	PSC	3	UC	55	5	620
PSC 10	41	Female	PSC	13	IC	14	8	236
Cholecystectomy 1	59	Female	None / Control				0	86
Cholecystectomy 2	41	Female	None / Control				0	41
Cholecystectomy 3	35	Male	None / Control				0	106

Patient	Antibiotic Use in the Last 3 Months	Antibiotics Day of Sample Collection	Corticosteroid Use in the Last 3 months	5-ASA	Biologics	UDCA	Proton Pump Inhibitors
PSC 1	Ciprofloxacin, vancomycin	Ciprofloxacin	Prednisone	Lialda	Stelara		
PSC 2	Vancomycin	Ciprofloxacin	Day of steroids (decadron) w/ anesthesia (8 mg IV)	Stopped mesalamine d/t clinical remission			
PSC 3	Augmentin, Vantin, Doxycycline	Piperacillin-tazobactam, discharged on bactrim				Yes	
PSC 4	Vantin, Levaquin	Piperacillin-tazobactam	Day of steroids (decadron) w/ anesthesia (4 mg IV)			Yes	
PSC 5	Ciprofloxacin, metronidazole	Ciprofloxacin		Pentasa		Yes	
PSC 6	Azithryomycin, ciprofloxacin, clindamycin (topical)	Ciprofloxacin (dosed twice)	Day of steroids (decadron) w/ anesthesia (4 mg IV)	Asacol		Yes	
PSC 7		Ciprofloxacin	Day of dexamethasone (4 mg IV)			No, trialed and did not tolerate	
PSC 8	Xifaxan, ciprofloxacin (x3 for UTIs over 3 months prior to ERCP)	Piperacillin-tazobactam		Mesalamine		Yes	

PSC 9		Ciprofloxacin	Acetonide 0.1 % ointment pre-procedure<br><br>Day of steroids (decadron) w/ anesthesia (4 mg IV)			Yes	Pantoprazole
PSC 10	Ciprofloxacin, metronidazole	Ciprofloxacin	Prednisone, Budesonide, Decadron (emergency ERCP), hydrocortisone rectal cream		Entyvio		Omeprezole
Chol. 1		Ceftriaxone					
Chol. 2		Ceftriaxone					
Chol. 3	Monodox, Bactroban (for acne)	Ceftriaxone					

Table S2. PSC 1 Colony 16S Sanger Sequencing and H_2S Production

Colony #	BLAST Result	H2S Production?	Notes
7	No priming	N	Query coverage, % ID, read length, CRL, QS information, and clonality groups are not available for PSC 1. Please see PSC 1 - 8 months for information from this patient.
8	No priming	N	
22	No priming	N	
18	Paraclostridium bifermentans/benzoelyticum	Y	
1	Streptococcus salivarius	N	Used in cell culture assays
2	Streptococcus salivarius	N	
3	Streptococcus salivarius	N	
4	Streptococcus salivarius	N	
5	Streptococcus salivarius	N	
6	Streptococcus salivarius	N	
9	Streptococcus salivarius	N	
10	Streptococcus salivarius	N	
11	Streptococcus salivarius	N	
12	Streptococcus salivarius	N	
13	Streptococcus salivarius	N	
14	Streptococcus salivarius	N	
15	Streptococcus salivarius	N	
16	Streptococcus salivarius	N	
17	Streptococcus salivarius	N	
19	Streptococcus salivarius	N	
20	Streptococcus salivarius	N	
21	Streptococcus salivarius	N	
23	Streptococcus salivarius	N	
24	Streptococcus salivarius	N	
25	Streptococcus salivarius	N	
26	Streptococcus salivarius	N	
27	Streptococcus salivarius	N	
28	Streptococcus salivarius	N	
29	Streptococcus salivarius	N	
30	Streptococcus salivarius	N	
31	Streptococcus salivarius	N	
32	Streptococcus salivarius	N	
33	Streptococcus salivarius	N	
34	Streptococcus salivarius	N	
35	Streptococcus salivarius	N	
36	Streptococcus salivarius	N	
37	Streptococcus salivarius	N	(Early sequencing termination)
38	Streptococcus salivarius	N	
39	Streptococcus salivarius	N	
40	Streptococcus salivarius	N	
41	Streptococcus salivarius	N	
42	Streptococcus salivarius	N	
43	Streptococcus salivarius	N	
44	Streptococcus salivarius	N	
45	Streptococcus salivarius	N	
46	Streptococcus salivarius	N	
47	Streptococcus salivarius	N	
48	Streptococcus salivarius	N	
49	Streptococcus salivarius	N	
50	Streptococcus salivarius	N	

Table S3. PSC 2 Colony 16S Sanger Sequencing and H_2S Production

Colony #	BLAST Result	H2S Production ?	NCBI BLAST Query Coverage	NCBI BLAST % ID	Read Length (raw)	Continuous Read Length (CRL)	Quality Score (QS)	Clonal group within the species	Notes
25	*No priming*	N	NA	NA	52	1	6	N/A	
39	*No priming*	N	NA	NA	52	1	11	N/A	
48	*No priming*	N	NA	NA	85	1	9	N/A	
5	Streptococcus anginosis OR Streptococcus sp. OR Streptococcus hominis	N	95	99.59	1033	939	47	N/A	
11	Streptococcus anginosis OR Streptococcus sp. OR Streptococcus hominis	N	91	99.28	1076	948	45	N/A	
12	Streptococcus anginosis OR Streptococcus sp. OR Streptococcus hominis	N	92	99.08	1053	952	46	N/A	
24	Streptococcus anginosis OR Streptococcus sp. OR Streptococcus hominis	N	96	98.39	1034	901	46	N/A	
21	Streptococcus sp.	N	98	98.01	1076	969	46	N/A	
27	Streptococcus sp.	N	95	98.16	1086	969	45	N/A	
29	Streptococcus sp.	N	95	98.17	1089	964	45	N/A	
34	Streptococcus sp.	N	99	97.69	1049	962	46	N/A	
36	Streptococcus sp.	N	97	98.84	1060	979	47	N/A	
37	Streptococcus sp.	N	97	98.74	1065	965	46	N/A	
4	Streptococcus salivarius	N	96	99.3	1032	965	47	1	
30	Streptococcus salivarius	N	92	99.19	1074	961	46	1	
31	Streptococcus salivarius	N	99	97.8	1057	975	46	1	
35	Streptococcus salivarius	N	93	99.02	1103	972	46	1	
38	Streptococcus salivarius	N	94	98.65	1103	962	45	1	
45	Streptococcus salivarius	N	91	99.3	1093	977	45	1	
1	Streptococcus salivarius OR Streptococcus sp.	N	98	99.3	1027	975	47	1	
6	Streptococcus salivarius OR Streptococcus sp.	N	98	99.11	1027	974	48	1	
7	Streptococcus salivarius OR Streptococcus sp.	N	98	98.69	1009	962	48	1	
20	Streptococcus salivarius OR Streptococcus sp.	N	97	98.57	1076	975	46	1	
23	Streptococcus salivarius OR Streptococcus sp.	N	94	99.5	1058	973	47	1	
42	Streptococcus salivarius OR Streptococcus sp.	N	97	98.81	1036	971	47	1	
44	Streptococcus salivarius OR Streptococcus sp.	N	95	97.37	1068	959	46	1	
2	Streptococcus salivarius OR Streptococcus sp. OR Streptococcus equinis	N	97	99.69	1024	905	47	1	
3	Streptococcus salivarius OR Streptococcus sp. OR Streptococcus equinis	N	95	98.78	1032	965	47	1	
8	Streptococcus salivarius OR Streptococcus sp. OR Streptococcus equinis	N	96	99.28	1006	949	48	1	
9	Streptococcus salivarius OR Streptococcus sp. OR Streptococcus equinis	N	97	98.71	1036	964	47	1	
10	Streptococcus salivarius OR Streptococcus sp. OR Streptococcus equinis	N	97	98.93	1062	979	47	1	
13	Streptococcus salivarius OR Streptococcus sp. OR Streptococcus equinis	N	97	98.83	1058	968	46	1	
14	Streptococcus salivarius OR Streptococcus sp. OR Streptococcus equinis	N	96	98.56	1076	973	46	1	
15	Streptococcus salivarius OR Streptococcus sp. OR Streptococcus equinis	N	86	98.68	1138	946	42	1	
16	Streptococcus salivarius OR Streptococcus sp. OR Streptococcus equinis	N	96	99.29	1022	966	47	1	
17	Streptococcus salivarius OR Streptococcus sp. OR Streptococcus equinis	N	95	99.29	1035	956	47	1	
18	Streptococcus salivarius OR Streptococcus sp. OR Streptococcus equinis	N	96	99.02	1064	965	46	1	
19	Streptococcus salivarius OR Streptococcus sp. OR Streptococcus equinis	N	93	99.3	1072	970	46	1	
22	Streptococcus salivarius OR Streptococcus sp. OR Streptococcus equinis	N	97	98.74	1063	973	47	1	
26	Streptococcus salivarius OR Streptococcus sp. OR Streptococcus equinis	N	94	99.39	1044	969	46	1	
28	Streptococcus salivarius OR Streptococcus sp. OR Streptococcus equinis	N	91	99.39	1075	960	45	1	
32	Streptococcus salivarius OR Streptococcus sp. OR Streptococcus equinis	N	95	98.68	1030	941	46	1	
33	Streptococcus salivarius OR Streptococcus sp. OR Streptococcus equinis	N	96	99.59	1021	956	47	1	
40	Streptococcus salivarius OR Streptococcus sp. OR Streptococcus equinis	N	95	98.98	1036	961	47	1	
41	Streptococcus salivarius OR Streptococcus sp. OR Streptococcus equinis	N	95	98.97	1014	938	46	1	
43	Streptococcus salivarius OR Streptococcus sp. OR Streptococcus equinis	N	97	98.75	1074	966	46	1	
46	Streptococcus salivarius OR Streptococcus sp. OR Streptococcus equinis	N	93	98.37	1058	954	45	1	
47	Streptococcus salivarius OR Streptococcus sp. OR Streptococcus equinis	N	94	99.09	1049	948	45	1	
49	Streptococcus salivarius OR Streptococcus sp. OR Streptococcus equinis	N	95	99.18	1034	954	45	1	
50	Streptococcus salivarius OR Streptococcus sp. OR Streptococcus equinis	N	94	99.09	1040	960	47	1	

Table S4. PSC 3 Colony 16S Sanger Sequencing and H_2S Production

Colony #	BLAST Result	H2S Production ?	NCBI BLAST Query Coverage	NCBI BLAST % ID	Read Length (raw)	Continuous Read Length (CRL)	Quality Score (QS)	Clonal group within the species	Notes
1	Klebsiella pneumoniae OR Klebsiella sp.	N	96	100	906	856	52	1	used in cell culture assays
2	Klebsiella pneumoniae OR Klebsiella sp.	N	96	100	900	853	52	1	
3	Klebsiella pneumoniae OR Klebsiella sp.	N	96	99.89	910	860	52	1	
4	Klebsiella pneumoniae OR Klebsiella sp.	N	96	99.44	930	877	51	1	
5	Klebsiella pneumoniae	N	95	99.78	938	884	51	1	
6	Klebsiella pneumoniae OR Klebsiella sp.	N	95	99.77	924	869	51	1	
7	Klebsiella pneumoniae OR Klebsiella sp.	N	96	99.66	929	875	51	1	
8	Klebsiella pneumoniae OR Klebsiella sp.	N	96	99.66	919	868	52	1	
9	Klebsiella pneumoniae	N	96	99.88	894	843	52	1	
10	Klebsiella pneumoniae OR Klebsiella sp.	N	96	99.88	901	848	52	1	
11	Klebsiella pneumoniae OR Klebsiella sp.	N	96	99.89	906	853	52	1	
12	Klebsiella sp.	N	96	99.89	918	867	52	1	
13	Klebsiella pneumoniae OR Klebsiella sp.	N	96	99.44	920	864	51	1	
14	Klebsiella pneumoniae OR Klebsiella sp.	N	96	99.77	924	873	51	1	
15	Klebsiella pneumoniae OR Klebsiella sp.	N	96	100	914	857	51	1	
16	Klebsiella pneumoniae OR Klebsiella sp.	N	96	99.77	914	853	51	1	
17	Klebsiella pneumoniae OR Klebsiella sp.	N	96	99.21	921	856	50	1	
18	Klebsiella pneumoniae OR Klebsiella sp.	N	96	99.88	897	846	52	1	
19	Klebsiella pneumoniae OR Klebsiella sp.	N	96	99.88	906	846	52	1	
20	Klebsiella pneumoniae OR Klebsiella sp.	N	96	100	916	866	52	1	
21	Klebsiella pneumoniae OR Shigella flexneri OR Klebsiella sp.	N	96	100	919	869	52	2	
22	Klebsiella pneumoniae OR Klebsiella sp.	N	96	99.89	915	852	51	1	
23	Klebsiella pneumoniae	N	95	99.77	921	863	51	1	
24	Klebsiella pneumoniae	N	96	99.66	910	855	51	1	
25	Klebsiella pneumoniae OR Klebsiella sp.	N	96	99.53	888	833	52	1	
26	Klebsiella pneumoniae OR Klebsiella sp.	N	96	100	894	841	53	1	
27	Klebsiella pneumoniae OR Klebsiella sp.	N	96	99.76	886	827	52	1	
28	Klebsiella pneumoniae OR Klebsiella sp.	N	95	99.88	909	850	52	1	
29	Klebsiella pneumoniae OR Klebsiella sp.	N	95	99.65	892	836	52	1	
30	Klebsiella pneumoniae OR Klebsiella sp.	N	96	99.88	889	836	52	1	
31	Klebsiella pneumoniae OR Klebsiella sp.	N	96	100	889	836	52	1	
32	Klebsiella pneumoniae OR Klebsiella sp.	N	96	99.88	886	834	52	1	
33	Klebsiella pneumoniae OR Klebsiella sp.	N	96	100	860	810	53	1	
34	Klebsiella pneumoniae OR Klebsiella sp.	N	96	100	868	814	53	1	
35	Klebsiella pneumoniae OR Klebsiella sp.	N	95	100	876	817	53	1	
36	Klebsiella pneumoniae OR Klebsiella sp.	N	96	99.88	885	834	52	1	
37	Klebsiella pneumoniae OR Klebsiella sp.	N	95	99.88	887	828	52	1	
38	Klebsiella pneumoniae	N	96	99.88	870	821	53	1	
39	Klebsiella pneumoniae OR Klebsiella sp.	N	96	99.88	891	830	52	1	
40	Klebsiella pneumoniae OR Klebsiella sp.	N	95	99.88	869	812	52	1	
41	Klebsiella pneumoniae OR Klebsiella sp.	N	95	99.75	837	769	53	1	
42	Klebsiella pneumoniae OR Klebsiella sp.	N	95	99.88	853	793	53	1	
43	Klebsiella pneumoniae OR Shigella flexneri OR Klebsiella sp.	N	95	100	844	796	53	2	
44	Klebsiella pneumoniae OR Klebsiella sp.	N	96	100	848	798	53	1	
45	Klebsiella pneumoniae OR Klebsiella sp.	N	96	99.88	846	784	53	1	
46	Klebsiella pneumoniae OR Klebsiella sp.	N	95	99.75	846	790	53	1	
47	Klebsiella pneumoniae OR Klebsiella sp.	N	96	100	857	796	52	1	
48	Klebsiella pneumoniae OR Klebsiella sp.	N	96	100	860	800	52	1	
49	Klebsiella pneumoniae OR Klebsiella sp.	N	95	99.87	818	754	53	1	
50	Klebsiella pneumoniae OR Klebsiella sp.	N	95	100	831	770	53	1	

Table S5. PSC 6 Colony 16S Sanger Sequencing and H_2S Production

Colony #	BLAST Result	H2S Production?	NCBI BLAST Query Coverage	NCBI BLAST % ID	Read Length (raw)	Continuous Read Length (CRL)	Quality Score (QS)	Clonal group within the species	Notes
27	No priming	N	NA	NA	930	23	12	N/A	
28	No priming	N	NA	NA	942	1	12	N/A	
42	No priming	N	NA	NA	954	38	14	N/A	
50	No priming	N	NA	NA	917	1	12	N/A	
22	*Lactococcus lactis*	N	99	99.79	980	952	52	N/A	
33	*Lactococcus lactis*	N	98	99.59	989	953	50	N/A	
46	*Lactococcus lactis*	N	98	99.49	994	968	51	N/A	
1	*Enterococcus faecalis OR Enterococcus sp.*	N	98	99.34	925	890	51	1	used in cell culture assays
2	*Enterococcus faecalis OR Enterococcus sp.*	N	98	99.89	956	929	52	1	
3	*Enterococcus faecalis OR Enterococcus sp.*	N	98	99.89	950	925	52	1	
4	*Enterococcus faecalis OR Enterococcus sp.*	N	98	99.67	930	901	53	1	
5	*Enterococcus faecalis OR Enterococcus sp.*	N	98	99.67	922	892	53	1	
6	*Enterococcus faecalis OR Enterococcus sp.*	N	98	99.89	923	898	53	1	
7	*Enterococcus faecalis OR Enterococcus sp.*	N	98	99.89	939	914	53	1	
8	*Enterococcus faecalis OR Enterococcus sp.*	N	99	99.78	932	908	51	1	
9	*Enterococcus faecalis OR Enterococcus sp.*	N	98	99.15	954	918	51	1	
10	*Enterococcus faecalis OR Enterococcus sp.*	N	99	99.89	965	941	52	1	
11	*Enterococcus faecalis OR Enterococcus sp.*	N	99	100	973	949	52	1	
12	*Enterococcus faecalis OR Enterococcus sp.*	N	99	99.68	961	933	52	1	
13	*Enterococcus faecalis OR Enterococcus sp.*	N	98	100	955	932	52	1	
14	*Enterococcus faecalis OR Enterococcus sp.*	N	98	100	961	934	52	1	
15	*Enterococcus faecalis OR Enterococcus sp.*	N	98	100	938	913	53	1	
16	*Enterococcus faecalis OR Enterococcus sp.*	N	99	99.78	934	911	53	1	
17	*Enterococcus faecalis OR Enterococcus sp.*	N	98	99.79	977	940	50	1	
18	*Enterococcus faecalis OR Enterococcus sp.*	N	99	99.59	986	960	51	1	
19	*Enterococcus faecalis OR Enterococcus sp.*	N	98	99.69	983	956	51	1	
20	*Enterococcus faecalis OR Enterococcus sp.*	N	98	99.79	991	966	51	1	
21	*Enterococcus faecalis OR Enterococcus sp.*	N	98	99.79	985	957	51	1	
23	*Enterococcus faecalis OR Enterococcus sp.*	N	98	99.68	964	939	52	1	
24	*Enterococcus faecalis OR Enterococcus sp.*	N	98	99.68	948	916	52	1	
25	*Enterococcus faecalis OR Enterococcus sp.*	N	98	100	979	950	51	1	
26	*Enterococcus faecalis OR Enterococcus sp.*	N	98	99.59	995	969	51	1	
29	*Enterococcus faecalis OR Enterococcus sp.*	N	99	99.8	1001	973	51	1	
30	*Enterococcus faecalis OR Enterococcus sp.*	N	98	99.49	999	973	51	1	
31	*Enterococcus faecalis OR Enterococcus sp.*	N	98	99.9	984	958	52	1	
32	*Enterococcus faecalis OR Enterococcus sp.*	N	98	99.15	955	915	49	1	
34	*Enterococcus faecalis OR Enterococcus sp.*	N	98	99.69	997	972	51	1	
35	*Enterococcus faecalis OR Enterococcus sp.*	N	98	99.9	1001	973	50	1	
36	*Enterococcus faecalis OR Enterococcus sp.*	N	98	100	1009	984	51	1	
37	*Enterococcus faecalis OR Enterococcus sp.*	N	99	99.6	1012	968	50	1	
38	*Enterococcus faecalis OR Enterococcus sp.*	N	98	99.6	1030	969	50	1	
39	*Enterococcus faecalis OR Enterococcus sp.*	N	98	99.9	977	953	51	1	
40	*Enterococcus faecalis OR Enterococcus sp.*	N	98	99.58	962	931	51	1	
41	*Enterococcus faecalis OR Enterococcus sp.*	N	98	99.69	988	954	51	1	
43	*Enterococcus faecalis OR Enterococcus sp.*	N	99	99.8	1008	983	51	1	
44	*Enterococcus faecalis OR Enterococcus sp.*	N	99	99.9	1010	986	51	1	
45	*Enterococcus faecalis*	N	98	99.6	1005	980	51	1	
47	*Enterococcus faecalis OR Enterococcus sp.*	N	99	99.49	988	960	51	1	
48	*Enterococcus faecalis OR Enterococcus sp.*	N	99	99.89	964	939	52	1	
49	*Enterococcus faecalis OR Enterococcus sp.*	N	98	99.38	981	951	51	1	

Table S6. PSC 7 Colony 16S Sanger Sequencing and H_2S Production

Colony #	BLAST Result	H2S Production?	NCBI BLAST Query Coverage	NCBI BLAST % ID	Read Length (raw)	Continous Read Length (CRL)	Quality Score (QS)	Clonal group within the species	Notes
28	No priming	N	NA	NA	56	1	8	N/A	
1	*Schaalia odontolytica*	N	97	98.01	882	838	27	N/A	
16	*Schaalia odontolyica OR Actinomyces sp. OR Schaalia sp.*	N	95	97.75	884	812	27	N/A	
23	*Schaalia odontolyica OR Actinomyces odontolyticus*	N	65	86.11	898	61	16	N/A	
8	*Actinomyces odontolyticus OR Schaalia odontolytica*	N	98	99.07	877	834	53	N/A	
18	*Actinomyces sp. OR Actinomcyes bouchesdurhonensis*	N	98	99.23	922	887	51	N/A	Because species level identification was not possible, clonality analysis was not performed.
26	*Actinomyces sp. OR Actinomcyesbouchesdurhonensis*	N	97	96.89	826	745	46	N/A	
4	*Actinomyces sp. OR Actinomyces bouchesdurhonensis*	N	98	99.11	914	880	53	N/A	
10	*Actinomyces sp. OR Actinomyces bouchesdurhonensis*	N	98	99.42	904	859	52	N/A	
41	*Actinomyces sp.*	N	96	99.89	911	873	52	N/A	
22	*Actinomcyes sp. OR Actinomyces odontolyticus OR Schaalia odontolytica*	N	96	94.47	924	746	24	N/A	
25	*Actinomcyes sp. OR Actinomyces odontolyticus OR Schaalia odontolytica*	N	95	97.29	927	849	27	N/A	
32	*Actinomyces sp. OR Actinomyces odontolyticus OR Schaalia sp. OR Schaalia odontolyltica*	N	93	98.56	898	817	29	N/A	
19	*Actinomcyes sp. OR uncultured Schaalia sp. clone*	N	97	99.23	944	904	51	N/A	
43	*Actinomyces sp. OR Uncultured Schaalia sp.*	N	97	99.14	944	852	42	N/A	
13	*Actinomyces oris OR Actinomyces sp.*	N	92	98.83	924	812	43	N/A	
47	*Schaalia sp. OR Actinomyces sp. OR Actinomyces odontolyticus*	N	97	99.22	915	878	51	N/A	
11	*Campylobacter concisus*	N	98	99.89	925	899	54	N/A	
6	*Campylobacter showae*	N	98	100	903	873	54	N/A	
33	*Gemella parahaemolysans OR Gemella haemolysans OR Gemella taiwanensis OR Gemella sp.*	N	96	100	910	873	53	N/A	
36	*Lachnospiraceae bacterium*	N	98	99.68	948	908	49	N/A	
3	*Prevotella melaninogenica*	N	78	97.74	849	530	34	N/A	
46	*Prevotella melaninogenica*	N	98	99.13	938	887	49	N/A	
17	*Streptococcus anginosus*	N	98	100	929	906	54	N/A	
20	*Streptococcus boyakuensis OR Streptococcus sp. OR Streptococcus symci OR Streptococcus pneumoniae OR Streptococcus mitis*	N	98	100	941	916	53	N/A	
2	*Streptococcus lingualis*	N	98	100	910	884	54	N/A	
27	*Streptococcus lingualis*	N	98	99.89	936	910	53	N/A	
35	*Streptococcus mitis OR Streptococcus sp.*	N	99	99.89	935	913	54	N/A	
44	*Streptococcus oralis OR Streptococcus infantis*	N	98	99.78	934	910	53	N/A	
50	*Streptococcus oralis OR Streptococcus infantis*	N	98	99.77	906	875	54	N/A	
31	*Streptococcus oralis OR Streptococcus sp. OR Streptococcus agalactiae OR Streptococucs toyakuensis OR Streptococcus vulneris*	N	99	99.89	914	888	53	N/A	
30	*Streptococcus parasanguinis OR Streptococcus lingualis*	N	98	99.89	938	912	53	N/A	
12	*Streptococcus salivarius*	N	54	75.25	921	54	16	N/A	
49	*Streptococcus salivarius*	N	98	100	916	892	54	N/A	
9	*Streptococcus salivarius OR Streptococcus sp.*	N	98	99.66	893	864	54	N/A	
40	*Streptococcus salivarius OR Streptococcus sp.*	N	99	99.89	903	881	54	N/A	
39	*Streptococcus sp.*	N	99	94.99	911	594	25	N/A	
21	*Streptococcus sp. OR Streptococcus mitis*	N	98	99.89	933	907	53	N/A	
29	*Streptococcus sp. OR Streptococcus mitis*	N	99	99.89	944	921	53	N/A	
34	*Streptococcus sp. OR Streptococcus mitis*	N	98	99.89	926	897	53	N/A	
7	*Streptococcus sp. OR Streptococcus parasanguinis*	N	98	99.54	882	858	54	N/A	
37	*Streptococcus toyakuensis OR Streptococcus sp. OR Streptococcus symci*	N	98	99.89	940	915	52	N/A	
42	*Streptococcus toyakuensis OR Streptococcus sp. OR Streptococcus symci OR Streptococcus pneumoniae OR Streptococcus mitis*	N	98	99.89	928	900	54	N/A	
45	*Streptococcus toyakuensis OR Streptococcus sp. OR Streptococcus symci OR Streptococcus pneumoniae OR Streptococcus mitis*	N	98	99.89	942	915	52	N/A	
5	*Veillonella parvula*	N	99	99.89	905	882	53	N/A	used in cell culture assays
14	*Veillonella dispar OR Veillonella sp.*	N	99	99.89	912	887	51	N/A	used in cell culture assays
15	*Veillonella dispar OR Veillonella sp.*	N	98	100	900	875	54	N/A	
24	*Veillonella dispar OR Veillonella sp.*	N	70	89.35	858	335	29	N/A	
38	*Veillonella dispar OR Veillonella sp.*	N	99	99.78	935	909	52	N/A	
48	*Veillonella dispar OR Veillonella sp.*	N	98	99.77	897	870	54	N/A	

Table S7. PSC 8 Colony 16S Sanger Sequencing and H_2S Production

Colony #	BLAST Result	H2S Production ?	NCBI BLAST Query Coverage	NCBI BLAST % ID	Read Length (raw)	Continuous Read Length (CRL)	Quality Score (QS)	Clonal group within the species	Notes
1	*No Priming*	*N*	NA	NA	70	1	11	N/A	
19	*No Priming*	*N*	NA	NA	73	1	8	N/A	
5	Streptococcus anginosus OR Streptococcus hominis	N	98	99.4	1019	985	50	N/A	
17	Campylobacter gracilis	N	97	99.49	1010	966	49	N/A	
44	Prevotella phocaeensis OR Prevotella sp.	Y	98	99.27	979	950	51	N/A	
2	Fusobacterium necrophorum OR Fusobacterium necrophorum subsp. funduliforme	Y	95	99.47	1003	918	48	1	used in cell culture assays
4	Fusobacterium necrophorum OR Fusobacterium necrophorum subsp. funduliforme	N	95	98.86	1012	931	45	1	
8	Fusobacterium necrophorum OR Fusobacterium necrophorum subsp. funduliforme	Y	96	99.69	998	929	48	1	
10	Fusobacterium necrophorum OR Fusobacterium necrophorum subsp. funduliforme	Y	95	99.68	988	920	47	1	
11	Fusobacterium necrophorum OR Fusobacterium necrophorum subsp. funduliforme	Y	95	99.68	985	931	50	1	
13	Fusobacterium necrophorum OR Fusobacterium necrophorum subsp. funduliforme	Y	96	99.58	993	939	49	1	
14	Fusobacterium necrophorum OR Fusobacterium necrophorum subsp. funduliforme	N	97	99.05	979	929	47	1	
18	Fusobacterium necrophorum OR Fusobacterium necrophorum subsp. funduliforme	Y	97	99.69	1005	956	50	1	
21	Fusobacterium necrophorum OR Fusobacterium necrophorum subsp. funduliforme	Y	96	99.57	974	919	50	1	
22	Fusobacterium necrophorum OR Fusobacterium necrophorum subsp. funduliforme	Y	98	99.26	964	915	51	1	
23	Fusobacterium necrophorum OR Fusobacterium necrophorum subsp. funduliforme	Y	97	99.58	989	933	50	1	
24	Fusobacterium necrophorum OR Fusobacterium necrophorum subsp. funduliforme	Y	96	99.79	1012	957	49	1	
25	Fusobacterium necrophorum OR Fusobacterium necrophorum subsp. funduliforme	Y	96	99.69	1002	946	50	1	
26	Fusobacterium necrophorum OR Fusobacterium necrophorum subsp. funduliforme	Y	96	99.69	1007	953	50	1	
31	Fusobacterium necrophorum OR Fusobacterium necrophorum subsp. funduliforme	Y	96	99.67	955	902	51	1	
34	Fusobacterium necrophorum OR Fusobacterium necrophorum subsp. funduliforme	Y	97	99.48	992	939	50	1	
35	Fusobacterium necrophorum OR Fusobacterium necrophorum subsp. funduliforme	Y	96	100	995	945	50	1	
36	Fusobacterium necrophorum OR Fusobacterium necrophorum subsp. funduliforme	Y	95	99.68	982	910	49	1	
38	Fusobacterium necrophorum OR Fusobacterium necrophorum subsp. funduliforme	Y	97	99.47	969	901	50	1	
41	Fusobacterium necrophorum OR Fusobacterium necrophorum subsp. funduliforme	Y	95	99.79	1002	931	49	1	
43	Fusobacterium necrophorum OR Fusobacterium necrophorum subsp. funduliforme	Y	96	99.37	988	923	50	1	
45	Fusobacterium necrophorum OR Fusobacterium necrophorum subsp. funduliforme	Y	97	99.47	966	913	45	1	
47	Fusobacterium necrophorum OR Fusobacterium necrophorum subsp. funduliforme	Y	97	99.68	973	923	51	1	
49	Fusobacterium necrophorum OR Fusobacterium necrophorum subsp. funduliforme	Y	97	99.37	983	930	51	1	
50	Fusobacterium necrophorum OR Fusobacterium necrophorum subsp. funduliforme	Y	96	99.47	979	916	49	1	
6	Klebsiella sp.	N	96	99.38	1000	929	45	N/A	Because species level identification was not possible, clonality analysis was not performed.
9	Klebsiella sp.	N	93	99.25	998	919	47	N/A	
29	Klebsiella sp.	N	96	99.46	963	891	50	N/A	
30	Klebsiella sp.	N	97	99.05	971	897	50	N/A	
32	Klebsiella sp.	N	95	99.04	993	920	49	N/A	
33	Klebsiella sp.	N	96	98.85	995	919	48	N/A	
37	Klebsiella sp.	N	96	99.34	955	882	50	N/A	
42	Klebsiella sp.	N	96	99.37	993	916	49	N/A	
48	Klebsiella sp.	N	96	99.24	968	893	49	N/A	
46	Klebsiella sp.	N	95	99.56	952	879	49	N/A	
3	Klebsiella variicola OR Klebsiella pneumoniae OR Enterobacter roggenkampii	Y	95	99.06	1008	933	35	1	
15	Klebsiella variicola OR Klebsiella pneumoniae OR Klebsiella sp.	N	96	99.47	983	910	50	1	
16	Klebsiella variicola OR Klebsiella pneumoniae OR Klebsiella sp.	N	95	95.99	997	673	31	1	
20	Klebsiella variicola OR Klebsiella pneumoniae OR Klebsiella sp.	N	96	99.47	981	906	49	1	
27	Klebsiella variicola OR Klebsiella pneumoniae OR Klebsiella sp.	N	97	99.28	1002	932	49	1	
28	Klebsiella variicola OR Klebsiella pneumoniae OR Klebsiella sp.	N	96	99.57	980	907	49	1	
39	Klebsiella variicola OR Klebsiella pneumoniae OR Klebsiella sp.	N	96	99.79	983	938	51	1	
40	Klebsiella variicola OR Klebsiella pneumoniae OR Klebsiella sp.	N	96	99.79	980	930	51	1	
7	Klebsiella variicola OR Klebsiella pneumoniae OR Klebsiella sp. OR Enterobacter roggenkampii	Y	96	98.77	1009	935	45	1	
12	Klebsiella variicola OR Klebsiella pneumoniae OR Klebsiella sp. OR Enterobacter roggenkampii	N	97	99.26	982	911	50	1	

Table S8. PSC 9 Colony 16S Sanger Sequencing and H_2S Production

Colony #	BLAST Result	H2S Production?	NCBI BLAST Query Coverage	NCBI BLAST % ID	Read Length (raw)	Continuous Read Length (CRL)	Quality Score (QS)	Clonal group within the species	Notes
13	Clostridium perfringens OR Clostridium sp.	Y	99	99.51	1032	964	50	N/A	
14	Clostridium perfringens OR Clostridium sp.	Y	76	85.93	1023	19	14	N/A	
7	Enterococcus gallinarum OR Bacterium FCC9 OR Enterococcus casseliflavus	N	97	99.17	985	956	38	N/A	
9	Enterococcus gallinarum OR Enterococcus casseliflavus OR Enterococcus innesii OR Enterococcus sp. OR Enterococcus canintestini	N	98	98.78	999	957	36	N/A	
38	Enterococcus gallinarum OR Enterococcus casseliflavus OR Enterococcus innesii OR Enterococcus sp. OR Enterococcus canintestini	N	98	97.52	1066	962	35	N/A	
11	Enterococcus gallinarum OR Enterococcus sp. OR Enterococcus casseliflavus OR Enterococcus innesii	N	97	98.02	1040	953	36	N/A	
39	Enterococcus gallinarum OR Enterococcus sp. OR Enterococcus casseliflavus OR Enterococcus innesii OR Enterococcus canintestini	N	95	98.72	1060	950	36	N/A	
40	Enterococcus casseliflavus	N	98	98.52	1034	925	36	N/A	
42	Enterococcus casseliflavus	N	96	97.53	1056	942	36	N/A	
8	Fusobacterium animalis	Y	98	99.49	998	966	50	N/A	
2	Klebsiella quasipneumoniae subsp. similipneumoniae	N	98	98.09	1011	945	49	N/A	
48	Klebsiella quasipneumoniae subsp. Quasipneumoniae OR Klebsiella pneumoniae	N	97	98.79	1027	981	48	N/A	
15	Klebsiella variicola subsp. variicola OR Klebsiella pneumoniae OR Klebsiella quasipneumoniae subsp. quasipneumoniae OR Klebsiella sp.	N	98	99.19	1005	969	49	N/A	
17	Klebsiella sp.	N	98	98.42	1035	939	48	N/A	
22	Klebsiella sp.	N	99	98.09	1058	948	48	N/A	
27	Klebsiella sp.	N	92	98.78	1066	944	47	N/A	
28	Klebsiella sp.	N	92	98.98	1061	944	47	N/A	
34	Klebsiella sp.	N	98	98.37	1063	955	47	N/A	
4	Klebsiella pneumoniae OR Klebsiella quasipneumoniae subsp. similpneumoniae OR Klebsiella sp. OR Enterobacteriaceae bacterium	N	98	98.99	1013	953	49	1	
6	Klebsiella pneumoniae	N	98	98.88	995	956	49	1	
18	Klebsiella pneumoniae subsp. Rhinoscleromatis OR Klebsiella pneumoniae OR Klebsiella sp.	N	98	98.62	1038	976	48	2	
20	Klebsiella pneumoniae	N	92	98.99	1085	947	47	1	
23	Klebsiella pneumoniae	N	97	97.14	1073	948	47	1	
25	Klebsiella pneumoniae OR Klebsiella sp. OR Klebsiella quasipneumoniae subsp. quasipneumoniae	N	92	99.7	1074	978	47	1	
26	Klebsiella pneumoniae	Y	99	99.38	1067	954	48		
30	Klebsiella pneumoniae OR Klebsiella quasipneumoniae subsp. quaispneumoniae	N	90	98.88	1090	952	47	1	
32	Klebsiella pneumoniae OR Klebsiella quasipneumoniae subsp. quasipneumoniae OR Klebsiella sp.	N	96	98.87	1023	928	47	1	
33	Klebsiella pneumoniae	N	97	98.8	1036	963	48	1	
37	Klebsiella pneumoniae	N	66	79.04	1120	105	17	N/A	Poor sequence so included in species but not clonal group
43	Klebsiella pneumoniae	N	98	98.1	1079	984	48	1	
46	Klebsiella pneumoniae OR Klebsiella quasipneumoniae subsp. Quasipneumoniae OR Klebsiella sp.	N	92	99.09	1074	948	47	1	
49	Klebsiella pneumoniae subsp. rhinoscleromatis OR Klebsiella variicola OR Klebsiella pneumoniae	Y	97	98.92	1053	981	49	2	used in cell culture assays
1	Streptococcus anginosus OR Streptococcus sp.	N	98	99.8	991	958	51	1	
3	Streptococcus anginosus OR Streptococcus sp.	N	98	99.11	1025	974	50	1	
5	Streptococcus anginosus OR Streptococcus sp.	N	99	96.6	1011	983	51	1	
10	Streptococcus anginosus OR Streptococcus sp.	N	98	99.8	1018	989	51	1	
12	Streptococcus anginosus OR Streptococcus sp.	N	96	98.47	1083	938	44	1	
16	Streptococcus anginosus OR Streptococcus sp.	N	99	99.59	1018	981	51	1	
19	Streptococcus anginosus OR Streptococcus sp.	N	92	99.9	1072	988	49	1	
21	Streptococcus anginosus OR Streptococcus sp.	N	98	99.33	1062	980	49	1	
24	Streptococcus anginosus OR Streptococcus sp.	N	97	99.61	1055	1006	50	1	
29	Streptococcus anginosus OR Streptococcus sp.	N	93	99.6	1072	984	49	1	
31	Streptococcus anginosus OR Streptococcus sp.	N	95	98.48	1104	982	48	1	
35	Streptococcus anginosus OR Streptococcus sp.	N	98	98.86	1080	996	49	1	
36	Streptococcus anginosus	N	91	99.22	1118	1000	48	1	
41	Streptococcus anginosus OR Streptococcus sp.	N	98	99.81	1049	1009	50	1	
44	Streptococcus anginosus OR Streptococcus sp.	Y	92	95.84	1102	517	27	N/A	Issue with sequence quality so included in species but not clonal group
45	Streptococcus anginosus OR Streptococcus sp.	N	97	98.39	1086	1000	49	1	
47	Streptococcus anginosus OR Streptococcus sp.	N	98	99.52	1051	1000	50	1	
50	Streptococcus anginosus OR Streptococcus sp.	N	98	98.76	1065	977	50	1	

Table S9. PSC 10 Colony 16S Sanger Sequencing and H_2S Production

Colony #	BLAST Result	H2S Production?	NCBI BLAST Query Coverage	NCBI BLAST % ID	Read Length (raw)	Continuous Read Length (CRL)	Quality Score (QS)	Clonal group within the species	Notes
29	Bacterium NLAE-zlG441	Y	99	98.8	1018	983	48	N/A	
39	Shigella flexneri	Y	97	95.66	1067	966	43	N/A	Sequencing issue in the middle of sequence
6	Shigella flexneri OR Escherichia coli	Y	98	97.58	1045	931	46	N/A	
8	Shigella flexneri OR Escherichia coli OR Escherichia fergusonii	Y	94	98.36	1098	946	46	N/A	
24	Shigella flexneri OR Escherichia coli OR Escherichia fergusonii	Y	96	97.61	1089	945	46	N/A	
21	Shigella flexneri OR Escherichia fergusonii	Y	93	98.74	1106	948	44	N/A	
15	Uncultured clone OR Escherichia coli OR Shigella sonnei	Y	94	98.16	1097	945	44	N/A	
26	Uncultured organism clone OR Escherichia coli	Y	97	97.83	1094	956	45	N/A	
42	Escherichia fergusonii OR Shigella flexneri OR Escherichia sp. OR Escherichia coli	Y	99	98.97	985	951	49	N/A	
13	Escherichia sp.	Y	94	97.34	1122	895	38	N/A	
36	Escherichia sp.	Y	97	98.28	1078	979	47	N/A	
30	Eschericha coli	Y	99	98.82	1028	939	47	1	
31	Eschericha coli	Y	98	98.15	1045	946	48	1	
1	Escherichia coli	Y	93	97.91	1129	993	43	1	used in cell culture assays
2	Escherichia coli	Y	94	99.13	1100	992	45	1	
3	Escherichia coli	Y	96	96.92	1087	1028	44	1	
4	Escherichia coli	Y	97	96.59	1053	899	43	1	
5	Escherichia coli	Y	98	96.63	1031	936	44	1	
14	Escherichia coli	Y	93	96.88	1096	882	42	1	
16	Escherichia coli	Y	96	96.17	1056	894	43	1	
17	Escherichia coli	Y	98	96.92	1027	896	44	1	
18	Escherichia coli	Y	97	99.21	1037	942	47	1	
20	Escherichia coli	Y	95	97.77	1084	968	44	1	
22	Escherichia coli	Y	94	97.69	1099	951	45	1	
25	Escherichia coli	Y	93	97.28	1111	941	41	1	
32	Escherichia coli	Y	98	97.73	1074	976	44	1	
33	Escherichia coli	Y	97	96.68	1082	932	45	1	
38	Escherichia coli	Y	97	97.39	1068	957	44	1	
40	Escherichia coli	Y	97	99.01	1038	981	47	1	
41	Escherichia coli	Y	97	98.88	1006	949	48	1	
43	Escherichia coli	Y	97	98.2	1025	932	47	1	
45	Escherichia coli	Y	98	97.01	1050	942	47	1	
49	Escherichia coli	Y	98	97.31	1054	951	45	1	
50	Escherichia coli	Y	99	98.91	1026	973	48	1	
44	Escherichia coli OR Bacterium ZH-4 OR Escherichia marmotae OR Escherichia fergusonii OR Shigella flexneri OR Shigella sonnei	Y	98	98.37	1060	946	47	1	
48	Escherichia coli OR Bacterium ZH-4 OR Escherichia marmotae OR Escherichia sp. OR Shigella flexneri OR Shigella sonnei	Y	98	97.88	1060	955	46	1	
35	Escherichia coli OR Escherichia fergusonii	Y	94	97.87	1096	955	44	1	
28	Escherichia coli OR Escherichia marmotae OR Escherichia fergusonii OR Escherichia sp.	Y	96	98.52	1050	949	47	1	
7	Escherichia coli OR Escherichia marmotae OR Escherichia fergusonii OR Escherichia sp. OR Shigella sonnei	Y	97	98	1068	948	46	1	
10	Escherichia coli OR Escherichia marmotae OR Escherichia fergusonii OR Escherichia sp. OR Shigella sonnei	Y	94	98.17	1108	992	45	1	
11	Escherichia coli OR Escherichia marmotae OR Escherichia fergusonii OR Escherichia sp. OR Shigella sonnei	Y	92	99.11	1106	982	46	1	
12	Escherichia coli OR Escherichia marmotae OR Escherichia fergusonii OR Escherichia sp. OR Shigella sonnei	Y	94	98.65	1108	947	47	1	
19	Escherichia coli OR Escherichia marmotae OR Escherichia fergusonii OR Escherichia sp. OR Shigella sonnei	Y	98	98.47	1061	942	46	1	
23	Escherichia coli OR Escherichia marmotae OR Escherichia fergusonii OR Escherichia sp. OR Shigella sonnei	Y	90	97.98	1101	948	41	1	
27	Escherichia coli OR Escherichia marmotae OR Escherichia fergusonii OR Escherichia sp. OR Shigella sonnei	Y	98	98.38	1070	1013	46	1	
34	Escherichia coli OR Escherichia marmotae OR Escherichia fergusonii OR Escherichia sp. OR Shigella sonnei	Y	95	98.35	1085	937	46	1	
46	Escherichia coli OR Escherichia marmotae OR Escherichia fergusonii OR Shigella sonnei OR Shigella flexneri OR Escherichia sp.	Y	97	98.93	1065	941	48	1	
9	Escherichia coli OR Shigella sonnei	Y	92	98.17	1120	990	43	1	
37	Escherichia coli OR Shigella sonnei OR Escherichia marmotae	Y	98	97.56	1088	996	45	1	
47	Escherichia coli OR Shigella sonnei OR Escherichia marmotae	Y	98	97.6	1066	954	46	1	

Table S10. PSC 1 (8 Months Later) Colony 16S Sanger Sequencing and H_2S Production

Colony #	BLAST Result	H2S Production?	NCBI BLAST Query Coverage	NCBI BLAST % ID	Read Length (raw)	Continuous Read Length (CRL)	Quality Score (QS)	Clonal group within the species	Notes
6	No priming	N	NA	NA	555	20	14	N/A	
21	No priming	N	NA	NA	881	1	8	N/A	
28	No priming	N	NA	NA	948	7	13	N/A	
29	No priming	N	NA	NA	67	1	8	N/A	
20	Poor quality	N	NA	NA	970	10	14	N/A	
25	Poor quality	N	NA	NA	959	116	19	N/A	
26	Poor quality	N	NA	NA	958	47	16	N/A	
32	Poor quality	N	NA	NA	820	25	11	N/A	
50	Poor quality	N	NA	NA	859	41	13	N/A	
16	Actinomyces sp. OR Actinomcyes odontolyticus OR Schaalia odontolytica	N	93	93.01	985	297	21	N/A	
9	Campylobacter concisus	N	97	99.47	979	929	50	N/A	
33	Campylobacter concisus	N	97	99.89	964	921	51	N/A	
4	Neisseria perflava OR Neisseria subflava	N	98	99.59	1008	958	46	N/A	
5	Staphylococcus capitis OR Staphylococcus epidermidis OR Staphylococcus sp. OR Staphylococcus caprae	N	98	99.39	1000	964	50	N/A	
19	Streptococcus parasanguinis	N	98	98.99	1008	962	48	N/A	
24	Streptococcus rubneri OR Streptococcus australis OR Streptococcus sp. OR Streptococcus koreensis OR Streptococcus pneumoniae OR Streptococcus sp.	N	94	99.89	975	900	48	N/A	
1	Streptococcus salivarius OR Streptococcus sp.	N	98	99.49	992	941	50	1	Note that clonal group 2 differs by just 1 base pair from group 1
2	Streptococcus salivarius OR Streptococcus sp.	N	96	99.58	996	942	42	1	
3	Streptococcus salivarius OR Streptococcus sp.	N	98	99.39	1001	965	50	2	
7	Streptococcus salivarius OR Streptococcus sp.	N	98	99.26	964	927	49	2	
8	Streptococcus salivarius	N	97	100	979	918	49	1	
10	Streptococcus salivarius OR Streptococcus sp.	N	98	99.39	994	947	50	1	
11	Streptococcus salivarius OR Streptococcus sp.	N	97	99.58	991	933	50	1	
12	Streptococcus salivarius OR Streptococcus sp.	N	99	99.18	983	949	50	1	
13	Streptococcus salivarius OR Streptococcus sp.	N	97	98.7	1028	968	48	1	
14	Streptococcus salivarius OR Streptococcus sp.	N	98	99.58	958	925	51	1	
15	Streptococcus salivarius OR Streptococcus sp.	N	98	99.36	957	912	45	2	
17	Streptococcus salivarius OR Streptococcus sp.	N	98	99.38	991	942	50	2	
18	Streptococcus salivarius OR Streptococcus sp.	N	96	99.9	1009	953	50	1	
22	Streptococcus salivarius	N	97	99.36	970	896	37	N/A	Lower sequence quality so included in species but not clonal group
23	Streptococcus salivarius	N	97	99.47	972	916	50	1	
27	Streptococcus salivarius OR Streptococcus sp.	N	98	99.16	974	923	50	1	
30	Streptococcus salivarius OR Streptococcus sp.	N	98	99.47	967	918	51	1	
31	Streptococcus salivarius OR Streptococcus sp.	N	98	99.37	968	915	51	1	
34	Streptococcus salivarius OR Streptococcus sp.	N	98	99.26	967	921	50	1	
35	Streptococcus salivarius	N	96	100	958	905	50	1	
36	Streptococcus salivarius OR Streptococcus sp.	N	98	99.25	953	901	50	1	
37	Streptococcus salivarius OR Streptococcus sp.	N	98	99.36	949	901	51	1	
38	Streptococcus salivarius OR Streptococcus sp.	N	98	99.47	957	903	50	1	
39	Streptococcus salivarius OR Streptococcus sp.	N	98	99.36	957	914	48	1	
40	Streptococcus salivarius OR Streptococcus sp.	N	98	99.46	949	903	50	1	
41	Streptococcus salivarius OR Streptococcus sp.	N	96	99.89	939	876	50	1	
42	Streptococcus salivarius OR Streptococcus sp.	N	98	99.46	943	892	51	1	
43	Streptococcus salivarius OR Streptococcus sp.	N	98	99.35	936	884	51	1	
44	Streptococcus salivarius OR Streptococcus sp.	N	96	99.77	917	871	51	1	
45	Streptococcus salivarius OR Streptococcus sp.	N	98	99.45	931	897	52	1	
46	Streptococcus salivarius OR Streptococcus sp.	N	98	99.67	927	887	50	1	
47	Streptococcus salivarius OR Streptococcus sp.	N	98	99.23	924	878	51	1	
48	Streptococcus salivarius OR Streptococcus sp.	N	98	99.23	930	881	46	2	
49	Streptococcus salivarius OR Streptococcus sp.	N	96	99.77	914	851	46	2	

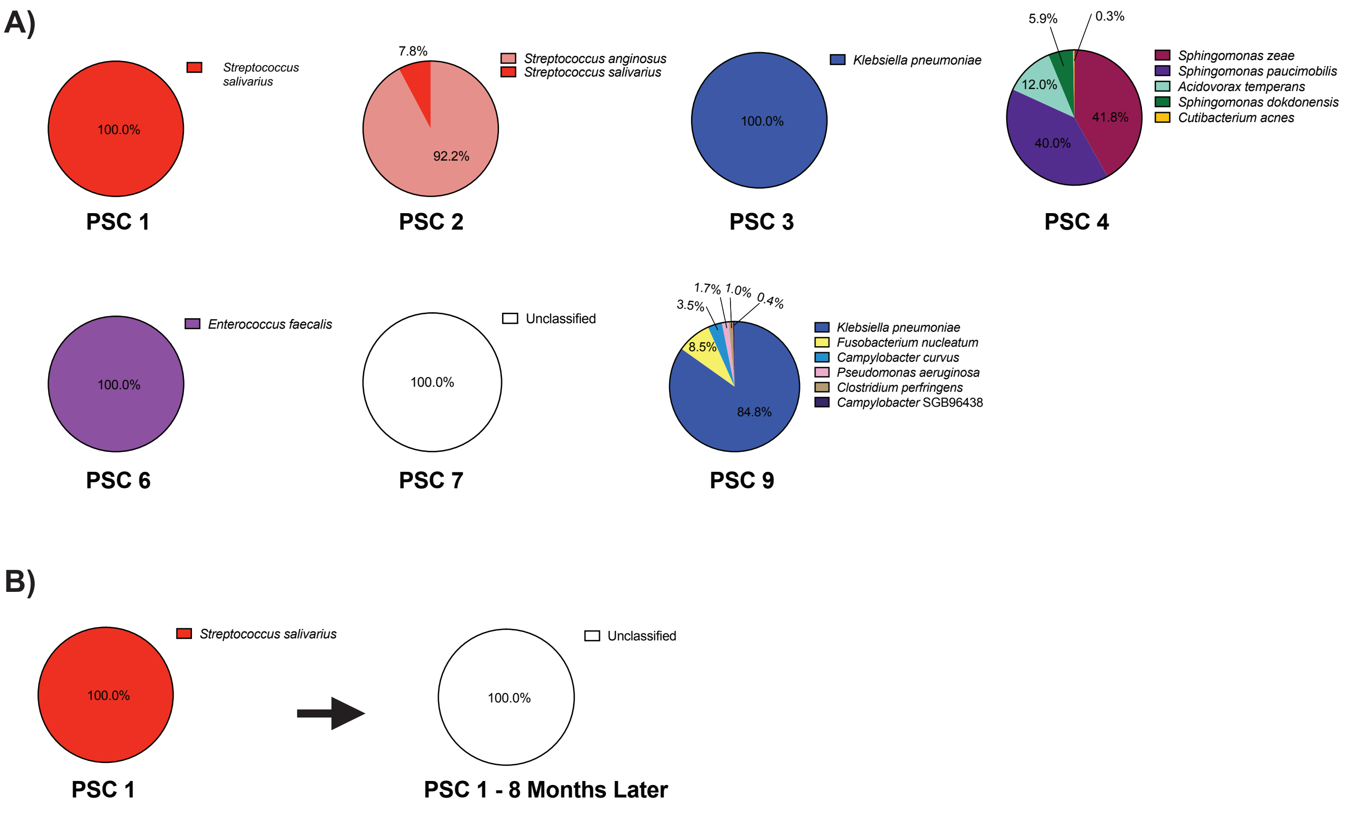

Figure S1. Relative abundance of bacterial taxa in human bile samples as determined by shotgun metagenomic sequencing. Each pie chart represents the microbial community composition of a distinct human bile sample. The relative abundances of each bacterial taxa are expressed as percentages of the total microbial community within each sample. Taxonomic profiling and relative abundances were determined using MetaPhlan 4.0 (1). NCBI BioProject database: PRJNA1169933.

Table S11. Source for bacteria used in cellular assays

	Source	Moniker in manuscript
Enterococcus faecalis	PSC 6, Colony 1	*E. faecalis*_PSC06_col01
Escherichia coli	PSC 10, Colony 1	*E. coli*_PSC10_col01
Fusobacterium necrophorum	PSC 8, Colony 2	*F. necrophorum*_PSC08_col02
Klebsiella pneumoniae **(H_2S producer)**	PSC 9, Colony 49	*K. pneumoniae*_PSC09_col49
Klebsiella pneumoniae **(non-producer)**	PSC 3, Colony 1	*K. pneumoniae*_PSC03_col01
Streptococcus salivarius	PSC 1, Colony 1	*S. salivarius*_PSC01_col01
Veillonella dispar	PSC 7, Colony 14	*V. dispar*_PSC07_col14
Veillonella parvula	PSC 7, Colony 5	*V. parvula*_PSC07_col05

Table S12. Relative abundance profiling of metagenomic data using Kraken2 (2) followed by KronaTools (3). NCBI BioProject database: PRJNA1169933.

	Bacteria (%)	Eukaryota (%)
**PSC 1**	3	88
**PSC 2**	18	71
**PSC 3**	24	63
**PSC 4**	18	69
**PSC 6**	18	71
**PSC 7**	0.4	91
**PSC 9**	58	24
**PSC 1 (8 months later)**	1	91

Table S13. Metagenomic sequencing statistics. NCBI BioProject database: PRJNA1169933.

PSC Sample	Total Read Pairs	Total Reads (R1 + R2)	Total bp > Q30	% bp > Q30
PSC 1	5.93E+07	1.19E+08	1.53E+10	93.973
PSC 1 – 8 months later	3.31E+07	6.63E+07	9.03E+09	91.553
PSC 2	3.68E+07	7.37E+07	1.01E+10	92.503
PSC 3	6.17E+07	1.23E+08	1.59E+10	93.886
PSC 4	3.88E+07	7.75E+07	1.07E+10	93.44
PSC 6	3.66E+07	7.32E+07	1.01E+10	92.335
PSC 7	2.76E+07	5.52E+07	7.51E+09	92.278
PSC 9	2.77E+07	5.53E+07	7.65E+09	92.756

Table S14. Metagenomic data NCBI BioProject database (PRJNA1169933) accession numbers for each sample.

	Accession #
**PSC 1**	SAMN44088305
**PSC 2**	SAMN44088306
**PSC 3**	SAMN44088307
**PSC 4**	SAMN44088308
**PSC 6**	SAMN44088309
**PSC 7**	SAMN44088310
**PSC 9**	SAMN44088311
**PSC 1 (8 months later)**	SAMN44088312

Table S15. Observed average OD_{600} after overnight culture (n = 6)

	Source	Mean OD600 after Normalized Overnight Culture	Standard Deviation OD600 after Normalized Overnight Culture
Enterococcus faecalis	PSC 6, Colony 1	1.75	0.10
Escherichia coli	PSC 10, Colony 1	1.76	0.06
Fusobacterium necrophorum	PSC 8, Colony 2	1.78	0.03
Klebsiella pneumoniae (H2S producer)	PSC 9, Colony 49	1.91	0.05
Klebsiella pneumoniae (non-producer)	PSC 3, Colony 1	1.72	0.21
Streptococcus salivarius	PSC 1, Colony 1	1.82	0.02
Veillonella dispar	PSC 7, Colony 14	0.29	0.05
Veillonella parvula	PSC 7, Colony 5	2.00*	0.00
Bacteroides fragilis	ATCC 25285	2.00*	0.00
Enterococcus gallinarum	Martin Kriegel, Yale School of Medicine	1.90	0.06

*OD_{600} of 2.00 is the maximum possible measurement.

Table S16. qPCR primers for human targets. Primer efficiencies were determined to be between 90 – 110% with R^2 values between 0.99 and 1.00.

	PrimerBank ID	Forward	Reverse
**Muc1**	324120973c1	TGCCGCCGAAAGAACTACG	TGGGGTACTCGCTCATAGGAT
**Muc5AC**	3334747a2	CCATTGCTATTATGCCCTGTGT	TGGTGGACGGACAGTCACT
**TNF-alpha**	25952110c2	GAGGCCAAGCCCTGGTATG	CGGGCCGATTGATCTCAGC
**IL-6**	224831235c1	ACTCACCTCTTCAGAACGAATTG	CCATCTTTGGAAGGTTCAGGTTG
**IL-8**	10834978a2	ACTGAGAGTGATTGAGAGTGGAC	AACCCTCTGCACCCAGTTTTC
**IL-17A**	27477085c1	TCCCACGAAATCCAGGATGC	GGATGTTCAGGTTGACCATCAC
**IFNg**	56786137c1	TCGGTAACTGACTTGAATGTCCA	TCGCTTCCCTGTTTTAGCTGC
**AE2**	314122223c1	TCCTCCCACCACATCCATCA	CTCCTCAATGGTCGGGGTTTC
**SUOX**	74099701c2	ACTCAAGTCAATCCCCTCAAGG	GCTGGAGTTATCACCAGAGAAGG
**TST**	34335291c2	GACTGGACTCGGGCCATATC	ACGTGGCAATGAGAGGCTG
**SQOR**	52851410c1	AGCTAGAGTGACTGAGTTGAACC	AGCTGGATTCCGAGAGCAATAA
**UBC**	305632811c1	CTGGAAGATGGTCGTACCCTG	GGTCTTGCCAGTGAGTGTCT

References

1. Blanco-Míguez A, Beghini F, Cumbo F, McIver LJ, Thompson KN, Zolfo M, Manghi P, Dubois L, Huang KD, Thomas AM, Nickols WA, Piccinno G, Piperni E, Punčochář M, Valles-Colomer M, Tett A, Giordano F, Davies R, Wolf J, ..., Segata N. 2023. Extending and improving metagenomic taxonomic profiling with uncharacterized species using MetaPhlAn 4. Nature Biotechnology 41:1633-1644.

2. Lu J, Rincon N, Wood DE, Breitwieser FP, Pockrandt C, Langmead B, Salzberg SL, Steinegger M. 2022. Metagenome analysis using the Kraken software suite. Nature Protocols 17:2815-2839.

3. Ondov BD, Bergman NH, Phillippy AM. 2011. Interactive metagenomic visualization in a Web browser. BMC Bioinformatics 12:385.
